# Glutamine synthetase regulates the immune microenvironment and cancer development through the inflammatory pathway

**DOI:** 10.7150/ijms.75625

**Published:** 2023-01-01

**Authors:** Do Thi Minh Xuan, Chung-Che Wu, Wei-Jan Wang, Hui-Ping Hsu, Hoang Dang Khoa Ta, Gangga Anuraga, Chung-Chieh Chiao, Chih-Yang Wang

**Affiliations:** 1 Graduate Institute of Cancer Biology and Drug Discovery, College of Medical Science and Technology, Taipei Medical University, Taipei 11031, Taiwan.; 2 Department of Neurosurgery, Taipei Medical University Hospital, Taipei 11031, Taiwan.; 3 Department of Surgery, School of Medicine, College of Medicine, Taipei Medical University, Taipei 11031, Taiwan.; 4 Research Center for Cancer Biology, China Medical University, Taichung 40676, Taiwan.; 5 Department of Surgery, National Cheng Kung University Hospital, College of Medicine, National Cheng Kung University, Tainan 70101, Taiwan.; 6 Ph.D. Program for Cancer Molecular Biology and Drug Discovery, College of Medical Science, Taipei Medical University, Taipei 11031, Taiwan.; 7 Department of Statistics, Faculty of Science and Technology, PGRI Adi Buana University, East Java, Surabaya 60234, Indonesia.; 8 TMU Research Center of Cancer Translational Medicine, Taipei Medical University, Taipei 11031, Taiwan.

**Keywords:** breast cancer, tamoxifen, glutamine synthetase (GLUL), immune microenvironment, bioinformatics

## Abstract

Although adjuvant tamoxifen therapy is beneficial to estrogen receptor-positive (ER^+^) breast cancer patients, a significant number of patients still develop metastasis or undergo recurrence. Therefore, identifying novel diagnostic and prognostic biomarkers for these patients is urgently needed. Predictive markers and therapeutic strategies for tamoxifen-resistant ER^+^ breast cancer are not clear, and micro (mi)RNAs have recently become a focal research point in cancer studies owing to their regulation of gene expressions, metabolism, and many other physiological processes. Therefore, systematic investigation is required to understand the modulation of gene expression in tamoxifen-resistant patients. High-throughput technology uses a holistic approach to observe differences among expression profiles of thousands of genes, which provides a comprehensive level to extensively investigate functional genomics and biological processes. Through a bioinformatics analysis, we revealed that glutamine synthetase/glutamate-ammonia ligase (*GLUL*) might play essential roles in the recurrence of tamoxifen-resistant ER^+^ patients. *GLUL* increases intracellular glutamine usage via glutaminolysis, and further active metabolism-related downstream molecules in cancer cell. However, how *GLUL* regulates the tumor microenvironment for tamoxifen-resistant ER^+^ breast cancer remains unexplored. Analysis of MetaCore pathway database demonstrated that *GLUL* is involved in the cell cycle, immune response, interleukin (IL)-4-induced regulators of cell growth, differentiation, and metabolism-related pathways. Experimental data also confirmed that the knockdown of *GLUL* in breast cancer cell lines decreased cell proliferation and influenced expressions of specific downstream molecules. Through a Connectivity Map (CMap) analysis, we revealed that certain drugs/molecules, including omeprazole, methacholine chloride, ioversol, fulvestrant, difenidol, cycloserine, and MK-801, may serve as potential treatments for tamoxifen-resistant breast cancer patients. These drugs may be tested in combination with current therapies in tamoxifen-resistant breast cancer patients. Collectively, our study demonstrated the crucial roles of *GLUL*, which provide new targets for the treatment of tamoxifen-resistant breast cancer patients.

## Introduction

According to the latest mortality data provided by the U.S. National Center for Health Statistics, the incidence and mortality of cancer increases with an estimated 1,9 million newly diagnosed cases and 600,000 deaths nationwide in 2022 1. To date, breast cancer remains one of most frequently diagnosed malignancies in the world 2. Several studies documented heterogeneity in breast cancer in terms of great diversity in both the clinical characteristics of patients and the behaviors of tumors. As a result, clinically applicable classifications of breast cancer, which are primarily based on molecular categories and histological patterns, are still undergoing upgrades 3, 4. Based on the expression of human epidermal growth factor receptor-2 (HER-2), and the two hormone receptors - estrogen receptor (ER) and progesterone receptor (PR), breast cancer is subcategorized into five subtypes. The estrogen-positive cancer comprises at most 80% of total patients 5, 6. For non-metastatic estrogen-positive (ER^+^) breast cancer patients, tamoxifen serves as an adjuvant therapy to reduce the risk of recurrence 7, 8. Previous clinical trials demonstrated the effect of tamoxifen therapy for patients after radical surgery, regardless of menopausal condition, the extent of metastasis, and nodal status 9, 10. However, a large number of patients develop intrinsic resistance against tamoxifen shortly after anti-estrogen therapy 11. To date, a significant number of alterations in molecular profiling leading to drug resistance are identified, highlighting the complexity of estrogen receptor signaling and as well as its involvement in crosstalk with other signaling pathways within breast tumors 12, 13. There have been several reports on the mechanism of tamoxifen resistance, but they were incomplete. Among them, patients with specific cytochrome polymorphisms were reported to present a higher ratio of tamoxifen resistance and an increased probability of recurrence, which lead to the incurability of advanced ER^+^ breast cancer 14, 15. On the other hand, excessive availability of glutamine not only induces the mechanistic target of rapamycin complex 1 (mTORC1) but also triggers tamoxifen-resistance when cooperating with stromal fibroblasts 16-18. In response to this situation, existing salvage therapies for tamoxifen-resistant (TR) patients have been well-designed, including fulvestrant, cyclin-dependent kinase 4/6 (CDK4/6) inhibitors, and histone deacetylase (HDAC) inhibitors 19-21. However, not all patients respond properly to these therapies 22-25.

Glutamate-ammonia ligase (*GLUL*), also named glutamine synthetase (*GLNS*), is the enzyme responsible for the *de novo* biosynthesis of glutamine from glutamate and ammonia in an ATP-dependent reaction 26. As a conditionally crucial amino acid and crucial nutrient required in culture medium, glutamine not only serves as a major source of nitrogen for the synthesis of proteins, nucleic acids, and macromolecules but also supports the redox status to maintain amino acid homeostasis in mammalian cells *in vitro* 27, 28. For those reasons, glutamine remains one of the major concerns over the last decades 29, 30. More shreds of evidence further reveal that glutamine impacts numerous signaling pathways that contribute to tumor proliferation, activating the mammalian target of rapamycin (mTOR) kinase, and autophagy 31-34. These findings explain the regulation of glutamine metabolism in tumors. However, explanations as to how *GLUL* regulates the immune microenvironment and cancer development through the inflammatory pathways are largely lacking.

Micro (mi)RNAs, a class of small single-stranded RNAs, belong to the noncoding (nc)RNA family which functions in inhibiting protein translation or degrading transcripts by binding to the 3'-untranslated region (UTR) of messenger (m)RNAs. In particular, recent studies report that miRNAs contributed to the progression of breast cancer after tamoxifen treatment. The mechanisms include the activation of estrogen receptor alpha (ERα), progression of the cell cycle, regulation of apoptosis, and controlling epithelial-to-mesenchymal transition 68-71. For these reasons, studies of differentially expressed miRNAs (DEMs) are similar to the research of differentially expressed genes (DEGs) to improve the susceptibility of tamoxifen against breast cancer and provide novel insights for clinical doctors.

Therefore, our study aimed to provide a comprehensive understanding of the mechanisms involved in tamoxifen-resistant ER^+^ breast cancer using both high-throughput technology and wet-lab approaches. Microarray-based mRNA and miRNA expression profiles of tamoxifen-resistant (TR) and tamoxifen-sensitive (TS) ER^+^ breast cancer patients were extracted from relevant datasets available at the Gene Expression Omnibus (GEO), followed by DEMs analyses and DEGs analyses, prediction of downstream target genes, and subsequently gene regulatory network to determine potential biomarkers associated with tamoxifen resistance. Finally, wet lab experiments were performed to validate the preceding analytical results.

## Material and Methods

### Cell Culture and Gene Knockdown

The MCF-7 human epithelial breast cancer cell line, characterized by the presence of the ER, PR, and glucocorticoid receptors, was a kind gift from Prof. Chun Hei Antonio Cheung of National Cheng Kung University (NCKU, Taiwan). MCF-7 cells were maintained in RPMI-1640 complete medium (Corning, Corning, NY, USA) supplemented with 10% fetal bovine serum (FBS, Avantor, USA) plus 1% penicillin/streptomycin (Corning, Corning, NY, USA), and were kept inside a humidified incubator under typical condition (37°C and 5% CO_2_). *GLUL* gene silencing was generated using a small hairpin (sh)RNA knockdown vector system. All of the shRNA vectors harboring puromycin and enhanced green fluorescent proteins, including two respective recombinant encoding human *GLUL* shRNA, and one non-target control (pLKO.1) shRNA against luciferase (shLuc), were constructed by the National RNAi Core Facility (Academia Sinica, Taiwan; http://rnai.genmed.sinica.edu.tw). The MCF-7 cells were seeded into six-well plates 24h prior to being incubated with three respective lipofectamine and shRNA vectors for 24h, in RPMI-1640 medium containing polybrene (8µg/mL). During an additional 48 hours of lipofectamine transfection, cells were subsequently maintained in a regular medium containing 10% FBS, and stable clones expressing shRNA were selected by constant treatment with puromycin (2µg/mL) from 72 hours onward. A non-target control (pLKO.1) shRNA against luciferase (shLuc) was employed as an expression control. At day 28 post-transfection with lipofectamine, the efficacy of gene silencing was further confirmed by a quantitative reverse-transcription polymerase chain reaction (RT-qPCR) and Western blotting in two populations of sh*GLUL*-transfected MCF-7 cells relative to vector control group.

### RT-qPCR, Western Blotting and Colony-Formation Assay

Total RNA extracted from stable clones of sh*GLUL*-transfected MCF-7 cells was isolated and purified using a GENEzol™ TriRNA Pure Kit (Geneaid Biotech, Taiwan) prior to being subjected to complementary synthesis using a PrimeScript Synthesis Kit (Takara Bio, Japan), with triplicate determinations. The RT-qPCR was performed using GoalBio SYBR green master mix (Hycell International, Taiwan) on a Roche Light Cycler 96 platform. Primer pair sequences targeting *GLUL* and glyceraldehyde 3-phosphate dehydrogenase (*GAPDH*) were provided by Origene (Rockville, USA) and were constructed by MDBio, Inc. The relative fold changes in expression of the *GLUL* gene were calculated by the delta-delta Ct (2^-∆∆Ct^) method after being normalized against the mRNA level of *GAPDH* as the housekeeping gene.

Total protein extractions derived from cell lysates of stable clones of sh*GLUL*-transfected MCF-7 and sh*GLUL*-transfected MCF-7 cells were subjected to Western blotting following the manufacturer's protocol (Bio-Rad Laboratories, Hercules, CA, USA). For subsequent analyses, an anti-GLUL (GTX109121) rabbit polyclonal antibody (polyAb) (GeneTex, Hsinchu, Taiwan) and anti-GAPDH (GTX124502) rabbit polyAb (GeneTex) were employed as primary antibodies.

For the colony-formation assay, MCF-7 cells of the three experimental groups were respectively seeded at the low density (1000 cells/well) into six-well plates for 2~3 weeks until macroscopic colonies had formed. The medium was discarded, and cells were fixed with absolute methanol fixation (20 min at room temperature), followed by an incubation with 2% methylene blue staining. The number of proper colonies formed was counted by a stereomicroscope (×100), and experiments were performed in triplicate data.

### Bioinformatics and Dataset Analyses

In this study, datasets containing molecular profiles of either ER^+^ and/or PR^+^ breast cancer patients treated with tamoxifen, regardless of their clinical outcomes (with or without recurrence), were collected from the GEO database. Differential mRNA and miRNA expression profiles of primary and relapsed ER^+^ breast tumors following tamoxifen treatment were extracted from two groups of datasets, GSE9893 combined with GSE7378, and GSE46823 combined with GSE83292 35-38. All raw data were extracted from the NCBI GEO database and subjected to the CLC Genomics Workbench v10.1 for subsequent analyses as previously described 39, 40. In order to explore alterations in expression patterns of miRNAs and mRNAs between the TR and TS populations, the top 10% of highly DEGs in TR patients were calculated as previously described 41, 42, accompanied by false detection rate (FDR)-adjusted *p*-value less than 0.05. A list of significant DEGs was imported into the Ingenuity Pathway Analysis (IPA) and MetaCore platform to construct related biological networks, biological processes (BPs), and diseases, with significant cutoff points for the enrichment of a pathway or an annotated gene group set to *p*<0.05 as mentioned above 43. TargetScan and miRWalk 2.0 are integrated database capable of providing more than 150 million human microRNA-target predictions. In the GSE83292 and GSE46823 datasets, the most miRNAs were downregulated or upregulated among TR ER^+^ breast cancer patients compared to TS ones. Finally, four down-regulated miRNAs and five common up-regulated between these two datasets, and then subjected to evaluation of the repressive strength of miRNA binding to its target mRNA, allowing producing target gene prediction. A miRmap score of > 95 was applied as the selection threshold for this analysis 44-46.

### Protein Network and Gene Set Enrichment Analysis (GSEA)

The Search Tool for the Retrieval of Interacting proteins (STRING) is a huge database consolidating more than twenty billion interactions among approximately sixty-seven million proteins of nearly fourteen thousand organisms 47. The STRING database (version 11.0) was leveraged to build up the protein-protein interacting (PPI) networks, including direct (physical) and indirect (functional) interactions based on the DEGs obtained from the previous analyses. The K-means clustering algorithm was applied to categorize proteins into different clusters. The GSEA was subsequently employed using the Bioconductor “DESeq2” and “fgsea” packages in R Studio software to identify upregulated gene sets between TR and TS breast cancer patients to determine groups of genes associated with the disease. A normalized enrichment score (NES) was first calculated, followed by the FDR with the purpose of controlling the false positive proportion. In particular, an FDR of <0.25 was set as the boundary criterion, while a nominal *p*-value of <0.05 and an NES of >1.5 were set as thresholds 48.

### Tumor Immune Infiltration Analysis

Tumor Immune Estimation Resource (TIMER) provides comprehensive and systematic analyses of tumor-infiltrating immune cells across various cancer types, specifically six immune cell populations across 31 tumors from The Cancer Genome Atlas (TCGA). In other words, this web server helps estimate differences in infiltration levels of lymphoid lineages (including B cells, cluster of differentiation-positive (CD4^+^) T cells, and CD8^+^ T cells) along with myeloid lineages (including neutrophils, macrophages, and dendritic cells (DCs)) in the tumor microenvironment (TME) compared to adjacent normal tissues, using the DiffExp module with default parameters. Expression scatterplots between a pair of given genes in specific cancer types were constructed according to the Spearman correlation and estimated statistical significance, adjusted for tumor purity if necessary 49. The seaborn package (Python) was used to create a heatmap as previously discussed 50, 51.

### Human Protein Atlas and Connectivity Map (CMap) Analysis

Human Protein Atlas provides high-resolution images exhibiting spatial distributions of proteins in normal human tissues and various cancer types 52. Expression profiles in breast tissues of GLUL at the protein level were examined by immunohistochemical (IHC) staining of fixed malignant tissues versus normal adjacent tissues that were labeled with antibodies against GLUL. The CMap database comprises interaction profiles of nearly 7000 human cell lines which were treated with 1300 US Food and Drug Administration (FDA)-approved compounds 53. The final gene list of interest was subjected to a computational pipeline generated by the CMap platform to evaluate the cellular effects of given compounds. Correlations between specific compounds and breast cancer were ranked by standardized connection scores, perturbation stability, and *p*-values.

### Statistical Analysis

Comparisons among groups of interest were performed by GraphPad Prism 5.0 (GraphPad Software, San Diego, CA, USA), using a one-way analysis of variance (ANOVA) and Tukey's multiple-comparison test. A *p*-value of <0.05 was considered statistically significant.

## Results

### Differentially Expressed genes and altered pathway in TR and TS Patients

**Figure 1:**
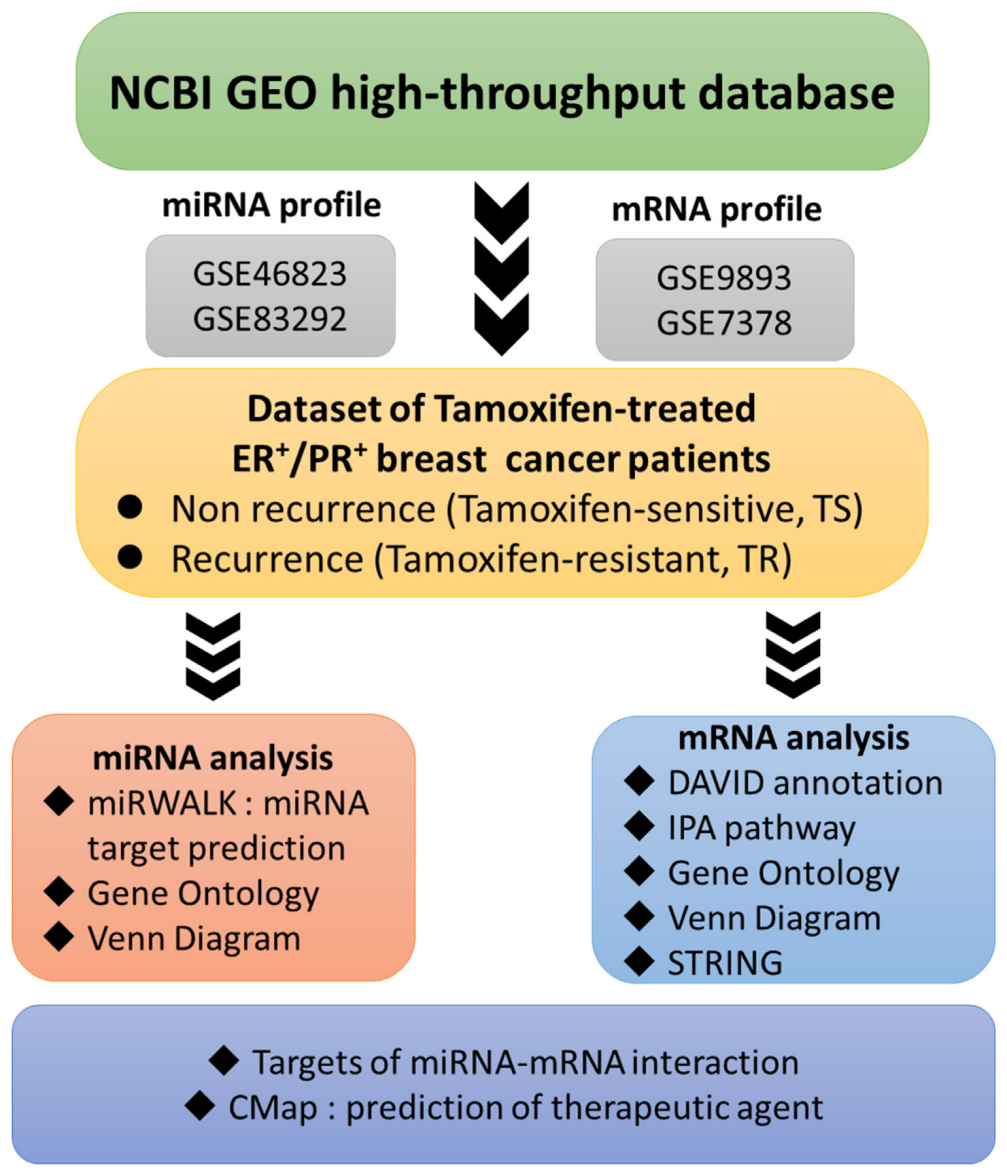
** Flow-chart illustrating the study design.** Raw data extracted from the NCBI GEO comprising micro (mi)RNA and messenger (m)RNA profiles of ER^+^ breast cancer patients were categorized into two groups according to their response to tamoxifen. **Abbreviations:** CMap, connectivity map; DAVID, database for annotation, visualization, and integrated discovery; ER, estrogen receptor; FDA, Food and Drug Administration; IPA, Ingenuity Pathway Analysis; TR, tamoxifen-resistant; TS, tamoxifen-sensitive.

We identified the differential expressions of mRNAs and miRNAs between TR and TS patients using miRtest, and evaluated individual miRNAs and their predicted target genes in the same analysis for verifying consistency. The up and down-regulated mRNAs and miRNAs were analyzed using numerous bioinformatics tools. The IPA was used to find upstream and downstream networks of miRNAs and mRNAs, STRING to build up PPI networks, and DAVID to investigate miRNA- and mRNA- associated functions and pathways. The miRWalk2.0, miRmap, and TargetScan were used to predict targets of the upregulated and downregulated miRNAs and to screen potential miRNA-mRNA interactions (Figure 1).

**Figure 2:**
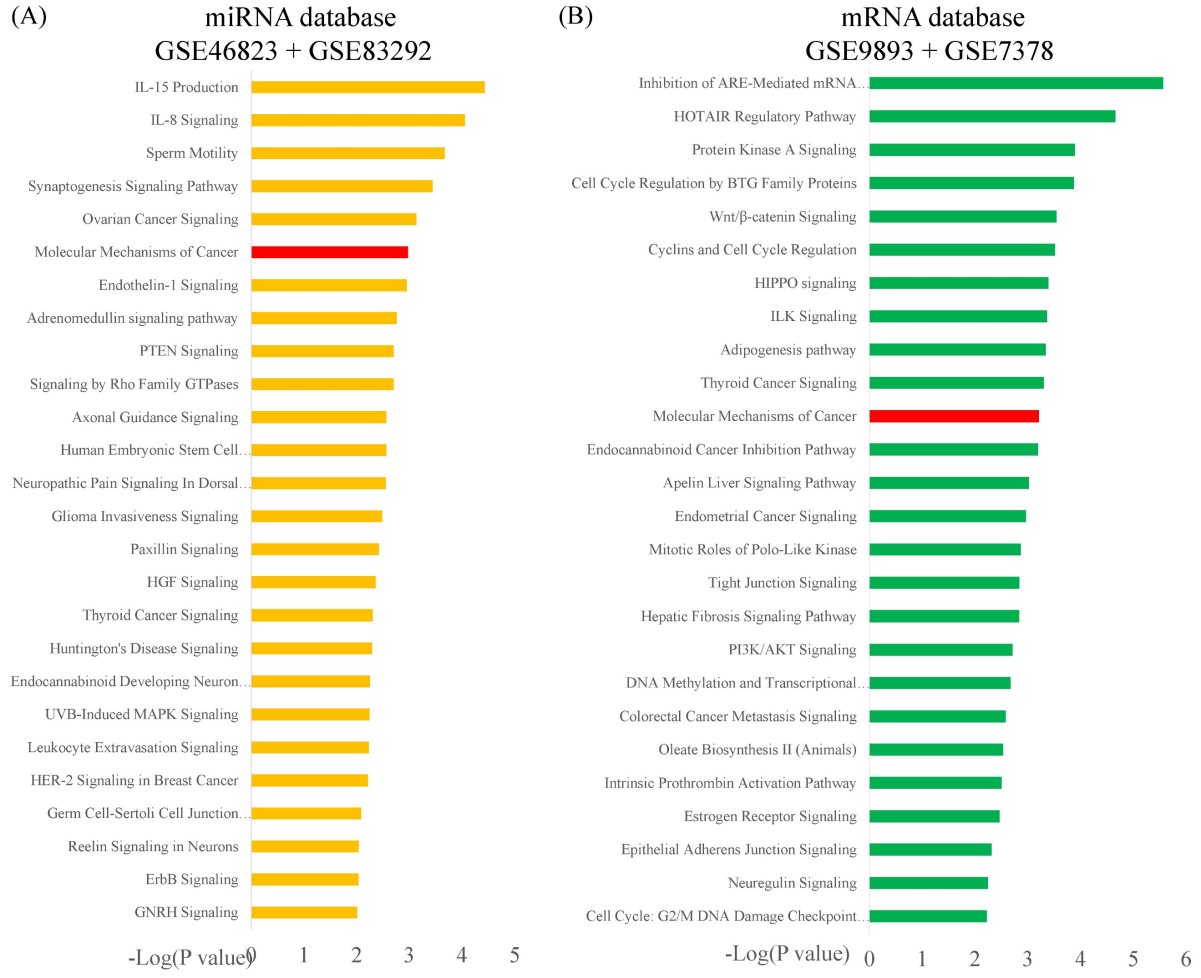
** Ingenuity Pathway Analysis (IPA): top key pathways associated with differentially expressed genes in estrogen receptor-positive (ER^+^) tamoxifen-resistant (TR) breast cancer patients, arranged in descending order of -log(*p-*value). (A)** IPA results of potential miRNA-targeted predicted genes in TR patients compared to TS patients. Data were collected from the GSE46823 and the GSE83292 datasets. **(B)** IPA results of the potential differentially expressed genes in TR patients compared to TS patients. Data were collected from the GSE9893 and the GSE7378 datasets. **Abbreviations:** miRNA, micro ribonucleic acid; ER^+^: estrogen receptor-positive. TR, tamoxifen-resistant; TS, tamoxifen-sensitive.

**Figure 3:**
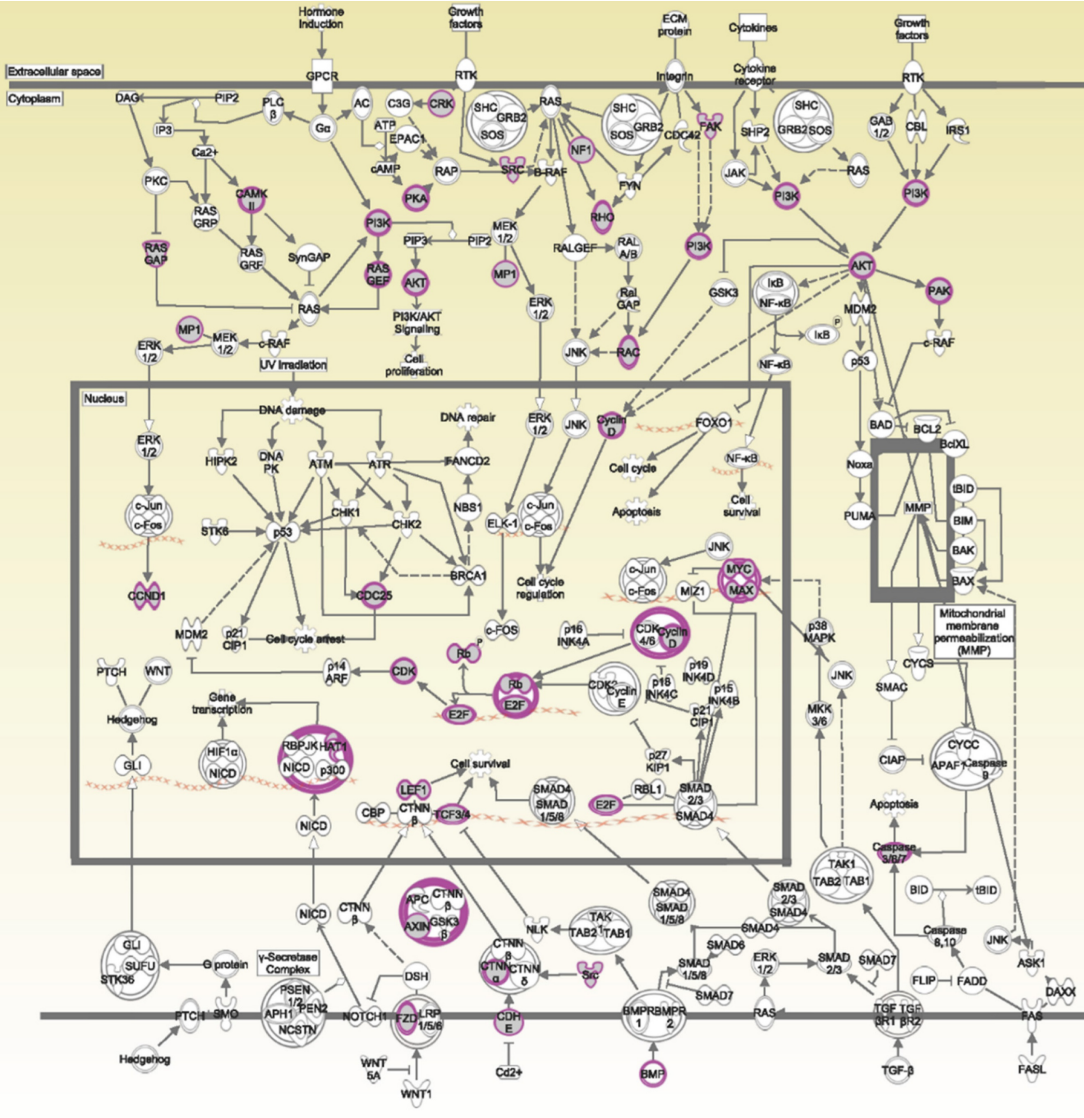
** Ingenuity Pathway Analysis (IPA): molecular mechanisms of cancer associated with differentially expressed genes in estrogen receptor-positive (ER^+^) tamoxifen-resistant (TR) breast cancer patients.** Purple boxes represent proteins involved in the common network. **Abbreviations:** ER^+^: estrogen receptor-positive. TR, tamoxifen-resistant; TS, tamoxifen-sensitive.

The potential genes for miRNA-mRNA interactions in TR patients were identified in the previous section, and the potential pathways and networks were explored in this section. The potential genes targeted by miRNA in GSE46823 and GSE83292 datasets were uploaded to the IPA. The canonical pathway was shown in Figure 2A and Supplementary Table S1. The “Molecular Mechanisms of Cancer” pathway was ranked as the top no.6 and included 14 target genes. Furthermore, the overlapping regulated genes in the GSE9893 and GSE7378 datasets were imported to the IPA and canonical pathways are in Figure 2B and Supplementary Table S2. Significantly enriched pathways included oncogenic pathways and regulation of the cell cycle. The “Molecular Mechanisms of Cancer” pathway was ranked in the top #11 and contained 10 upregulated genes. The detail of the “Molecular Mechanisms of Cancer” pathway was displayed in Figure 3. These oncogenic pathways play an essential role in genetic instability, carcinogenesis, and progression of ER^+^ TR breast cancer. Multiple oncogenes and tumor suppressor genes are contained in this canonical pathway, including G-protein-coupled receptors (GPCR) signaling, RAS/integrin signaling, AKT signaling, transforming growth factor (*TGF*)-*β*/bone morphogenetic protein (*BMP*) signaling, WNT signaling, Notch and Hedgehog (Hh) signaling, and death receptor signaling (the membranous receptor and intracellular part of Figure 3). These pathways were also associated with the expression of recombination signal binding protein for immunoglobulin kappa J region (RBPJ-κ), p300, histone acetyltransferase type B catalytic subunit (HAT1), and hypoxia-inducible factor 1α (*HIF1α*). The resulting three clusters with a core cluster containing all genes were related to cancer progression and metastasis.

### Gene Set Enrichment and Pathway Analysis of Tamoxifen-Treated Breast Cancer Patients

**Figure 4:**
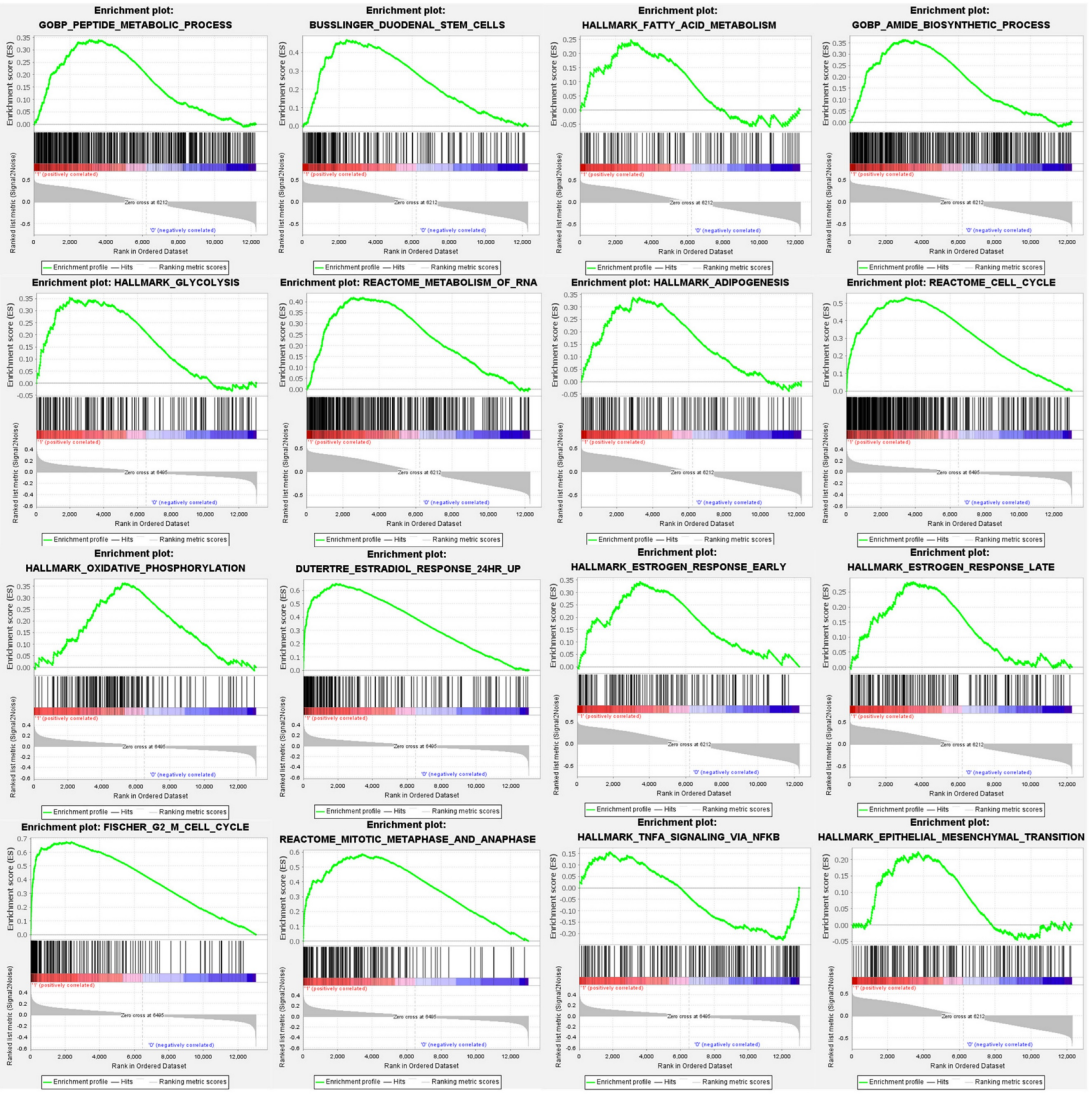
** Gene Set Enrichment Analysis (GSEA) enrichment curves of highly expressed gene sets in estrogen receptor-positive (ER^+^) tamoxifen treated breast cancer patients.** Positive enrichment scores (ER) reflect gene sets enriched at the top of the ranked list, with each bar indicating one specific gene located in the ranking. In each plot, bars on the far left in red represent positive correlations with the most highly upregulated genes, while bars on the far right in blue represent positive correlations with the most highly downregulated genes. The running sum of the weighted enrichment score in each gene set is denoted by the respective green curve. Statistical significance was set as follows: false discovery rate (FDR) <0.25, normalized enrichment score (NES) >1.5 and nominal p value <0.05.

For comprehensive exploration, we utilized a public database, the Gene Set Enrichment Analysis (GSEA), to verify the importance of targeted pathways in tamoxifen-treated primary breast cancer patients compared to controls. Upregulated genes were analyzed to retrieve the Gene Ontology enrichment results and Kyoto Encyclopedia of Genes and Genomes (KEGG), including cellular components (CCs), biological process (BPs), and molecular functions (MFs). The potentially regulated networks identified included peptide metabolic process, reactome cell cycle, fatty acid metabolism, adipogenesis, stem cells, epithelial-mesenchymal transition, estradiol response, oxidative phosphorylation, estrogen response late, G2 M cell cycle, glycolysis, IL-4 signaling, TNFA signaling via nuclear factor (NF)-κB, mitotic metaphase, and anaphase (Figure 4).

**Figure 5:**
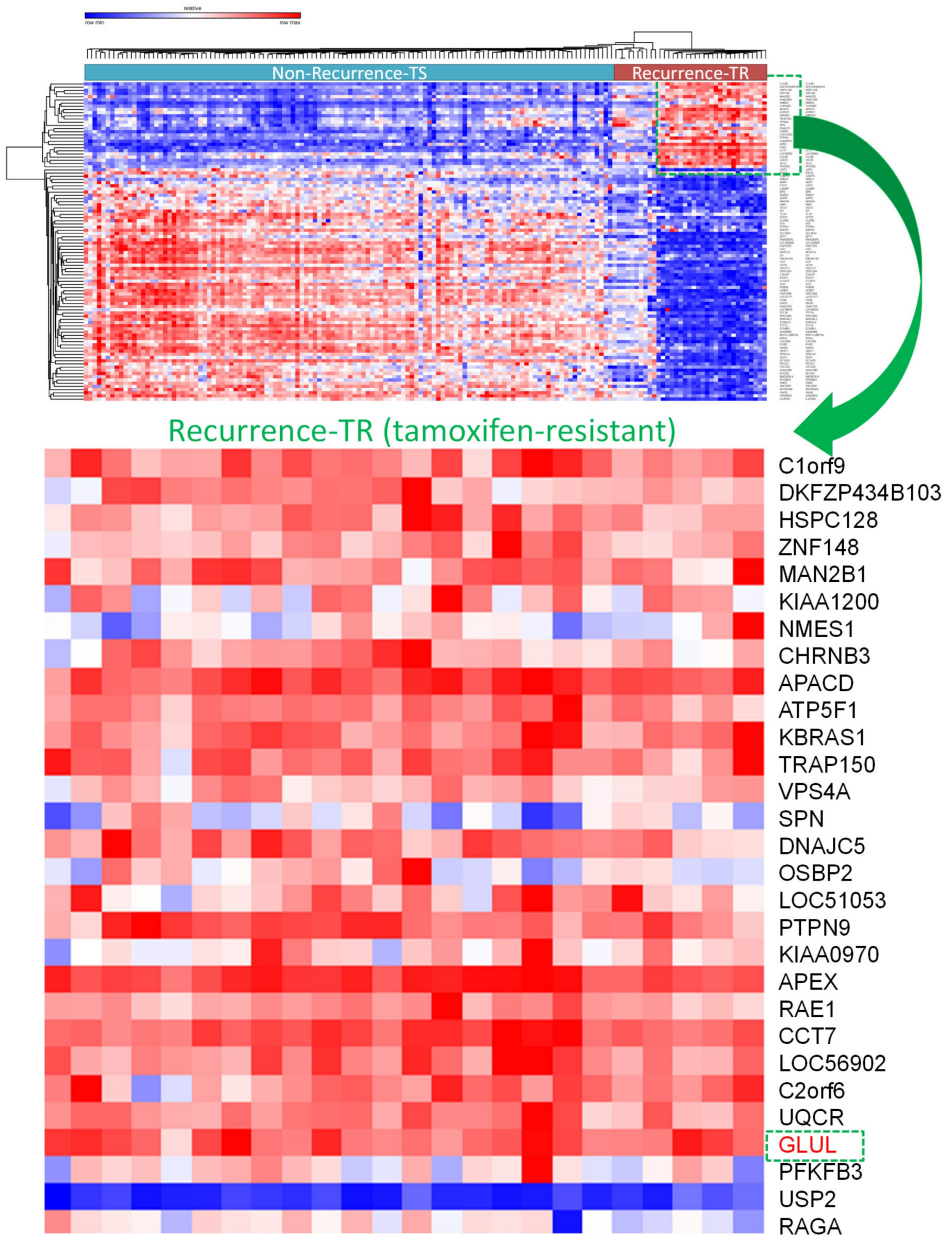
** Overview of differentially expressed genes in tamoxifen-sensitive (TS) and tamoxifen-resistant (TR) estrogen receptor-positive (ER^+^) breast cancer patients visualized in a heatmap format.** *GLUL* (glutamate-ammonia ligase) was one of the significantly upregulated genes in TR ER^+^ compared to TS ER^+^ breast cancer patients.

A selection of the most highly and least expressed miRNAs in TR patients compared to TS patients were extracted. There were 227 down-regulated miRNAs in the GSE46823 dataset and 157 miRNAs in the GSE83292 dataset. The intersection of these two groups obtained four down-regulated miRNAs: hsa-miR-1323, hsa-miR-711, hsa-miR-4287, hsa-miR-650. Whereas, there were 221 upregulated miRNAs in the GSE46823 dataset and 154 miRNAs in the GSE83292 dataset. The intersection of these two groups obtained five upregulated miRNAs: hsa-miR-532-5p, hsa-miR-1180, hsa-miR-152, hsa-miR-578, and hsa-miR-128. The TargetScan software was used for miRNA-targeting genes for each miRNA and these results were merged 54. Next, two mRNA datasets (GSE9893 and GSE7378) with ER^+^ breast cancer patients undergoing adjuvant tamoxifen therapy were selected. The top 10% of upregulated genes in TR compared to TS patients were selected. There were 1570 upregulated genes in GSE9893 and 1842 genes in GSE7378. The intersection of these genes obtained 167 common genes, which were extracted. A Venn diagram was employed to explore the genes shared by the aforementioned miRNA-targeting genes predicted by TargetScan and 167 upregulated genes obtained from previous DEGs analysis. Final results including significantly up-regulated genes in TR ER+ breast cancer patients compared to TS ER+ ones were presented in a heatmap (Figure 5). Among them, *GLUL* (glutamate-ammonia ligase) was selected for further analyses (Figure 5).

### GLUL Play an Important Role in Breast Cancer Development and Involved in Immune Regulation

**Figure 6:**
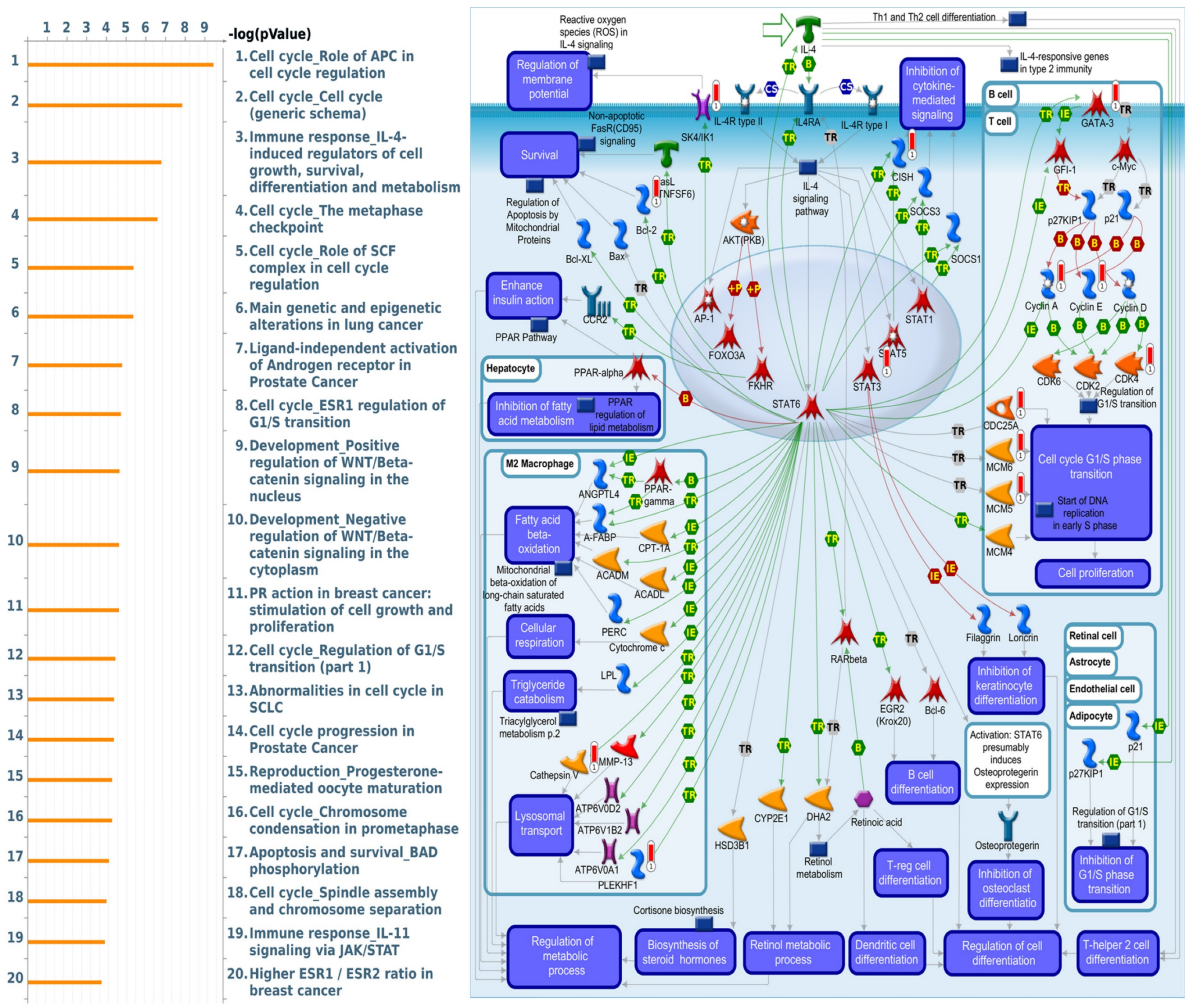
** Altered signaling pathways regulated by the top genes co-expressed with GLUL (glutamate-ammonia ligase) predicted by MetaCore.** The top 10% of genes co-expressed with GLUL from the METABRIC and TCGA databases were subjected to a Venn diagram to get a list of 943 genes in common, which subsequently underwent “biological processes” provided by MetaCore for downstream pathway analyses. The involved pathways were ranked in order of decreasing -log[p values]. “Immune response_IL-4-induced regulators of cell growth, survival, differentiation, and metabolism” was noteworthy for its third place on the list.

**Figure 7:**
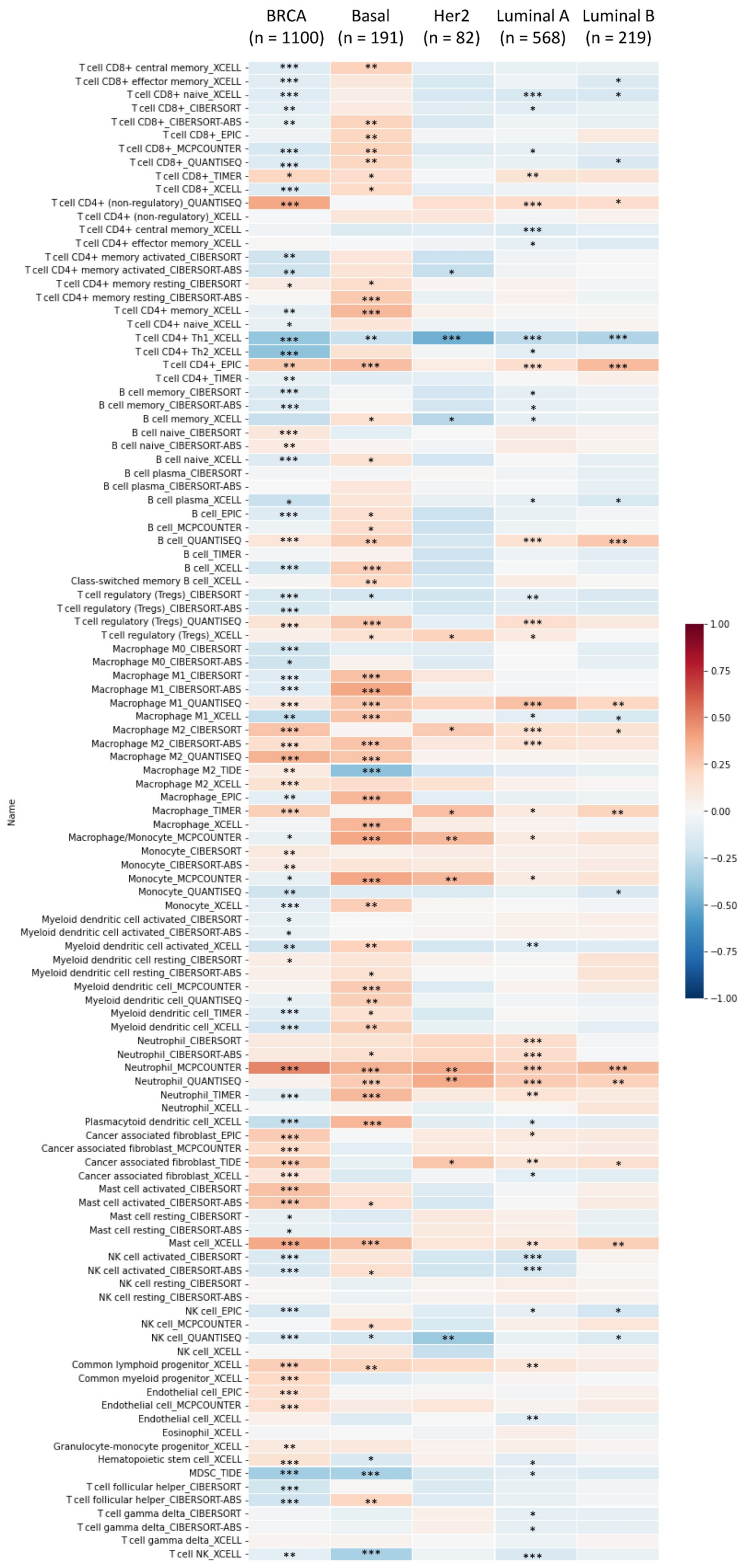
** Immune infiltration patterns of breast tumors in relation to glutamate-ammonia ligase (GLUL) estimated by TIMER visualized in a heatmap format.** Using the default setting of TIMER, correlations of expression levels of six major immune cell populations with four subtypes of breast cancer were evaluated based on a partial Spearman's rho, also referred to as Pearson correlation coefficient (*r*) that ranges from -1 (negative correlation) to +1 (positive correlation). *P < 0.05, **P < 0.01, and ***P < 0.001.

MetaCore is commonly employed to build up pathway networks from the input genes to simulate biological processes. We found interesting results related to GLUL, such as “Cell cycle_Role of APC in cell cycle regulation”, Immune response_IL-4-induced regulators of cell growth, survival, differentiation and metabolism”, “Cell cycle_role of SCF complex in cell cycle regulation”, “Cell cycle_ESR1 regulation of G1/S transition”, and “Apoptosis and survival_BAD phosphorylation”. These are immune- or cell-cycle-related pathways in the progression of breast cancer. The pathway lists and networks were respectively shown in Figure 6 and Supplementary Table S3. We found that the immune-related pathways were correlated with *GLUL* expression in breast cancer patients from public breast cancer patient datasets. IL-4 stimulates cell population proliferation through the activation of several cyclin-dependent kinases (CDKs), which promote the cell cycle G_1_/S phase transition. The increasing amount of information regarding the IL-4 pathway proved the importance of IL-4 in regulating metabolic processes. Meanwhile, an evolving tumor microenvironment is a convoluted and continuously growing entity. The configuration of the tumor microenvironment varies among tumor types. Several research reveal the important role of tumor microenvironment in cancer progression. We further evaluated the correlation coefficient between *GLUL* expression in breast cancer cells and activation of tumor-infiltrating immune cells, including B cells, M1 macrophages, M2 tumor-associated macrophages (M2 TAMs), neutrophils, and dendritic cells. A higher level of correlation coefficient was shown in red color, and these data suggested that *GLUL* expression was positively correlated with the function of immune cells (Figure 7). According to our knowledge, these data are the first to reveal the relationship between *GLUL* expression and subtypes of tumor-infiltrating immune cells.

**Figure 8:**
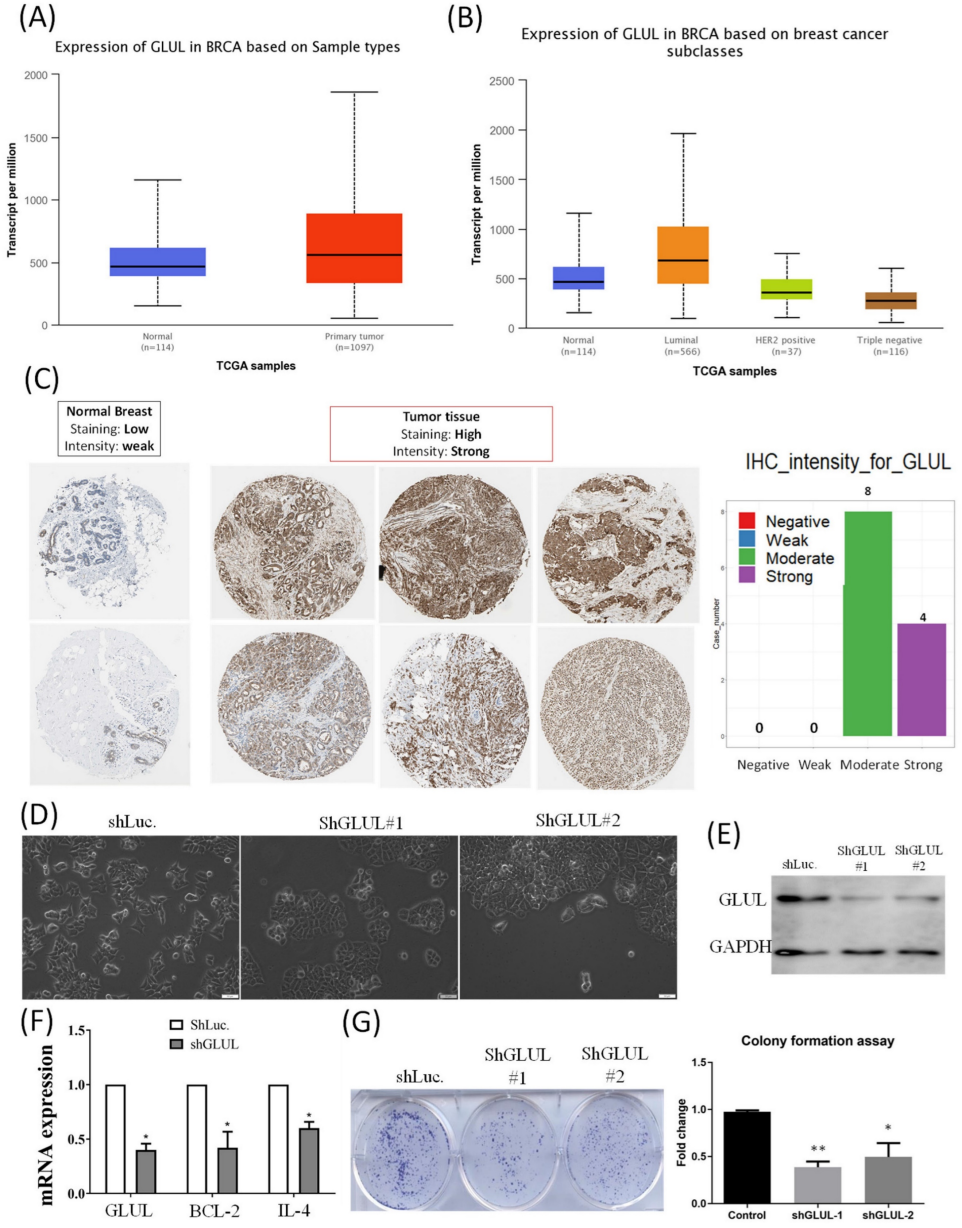
***GLUL* (glutamate-ammonia ligase) mRNA and protein in breast cancer patients and cell lines. (A)** Elevated *GLUL* expression at transcriptomic level in breast cancer compared to normal tissues. **(B)** Transcriptomic expression of *GLUL* in the different subtypes of breast cancer, which is up-regulated in the luminal subtype (ER^+^ cancer). **(C)** IHC staining of GLUL protein in breast cancer, but not in normal tissues. **(D)** Bright-field images of MCF7-shLuc as control, along with two *GLUL*-knockdown cell lines in two-dimensional cell cultures. **(E)** The efficacy of GLUL knockdown was further confirmed by Western blotting. **(F)** mRNA expression levels of downstream signaling pathways regulated by GLUL, including Bcl-2 which serves as a marker of the cell cycle, and IL-4 which is an immune-related marker. A pairwise comparison was made between the control cell line and GLUL-knockdown lines. **(G)** Colony-formation assay confirmed the influence of GLUL on suppressing the proliferation of MCF7 cells. The long-term proliferation of two MCF7 GLUL-knockdown cells were markedly declined compared to the MCF7-shLuc cell line as the control.

We analyzed transcripts of GLUL in the TCGA database. Expression of GLUL was higher in breast cancer samples compared to normal breast, especially in the ER^+^ breast cancer patients (Figure 8A, B). Expression patterns of *GLUL* protein in human breast cancer tissues were further confirmed by immunohistochemistry (IHC) staining provided by the Human Protein Atlas database. Staining levels of *GLUL* were increased in cancer specimens compared to normal breast (Figure 8C). We also investigated *GLUL* expression in various other types of cancer via the TCGA database (Supplementary Figure S1) and Human Protein Atlas (Supplementary Figure S2) databases. Results of bioinformatics analyses also revealed the molecular subtype specific for each cell line (Supplementary Figure S3), as well as DNA methylation levels of *GLUL* in breast cancer (Supplementary Figure S4). We confirmed that *GLUL* is important for the progression of ER^+^ breast cancer. Therefore, we chose the ER^+^ cell line- MCF-7 for further study. Lipofectamine transfection of *GLUL*-shRNA was performed to inhibit GLUL expression in MCF-7 cells. Intriguingly, *GLUL*-knockdown MCF-7 cells became more cuboidal shape in comparison with shLuciferase control cells, which is the typical morphology of epithelial-like luminal cancer (Figure 8D). The suppressive efficacy was confirmed by Western blotting (Figure 8E). Meanwhile, the expression levels of cell cycle- and immune-related markers decreased after downregulating GLUL expression, such as BCL-2 and IL-4 (Figure 8F). Specifically, IL-4 is a pleiotropic cytokine in both immune and non-immune cells regulating cell differentiation, survival, and proliferation (Supplementary Figure S5). Besides, the ability of anchorage-independent growth was suppressed in *GLUL*-knockdown MCF7- cells (Figure 8G). These results indicate that *GLUL* played a significant role in the inhibition of cellular proliferation and growth in breast epithelial cell.

### Identification of Potential Inhibitory Compounds from CMap

Up- and downregulated genes determined from comparisons between TR and TS patients through a Venn diagram in Figure 2 were uploaded to a CMap database to predict potential drugs for TR patients (Figure S6A). We selected the results of a drug sensitivity test from the MCF-7 ER^+^ cell line in the CMap database and compared these with the gene signatures of TR patients. The top 25 compounds with negative correlations were ranked by *p* values. Fulvestrant ranked number 4 (from -1 to 1), and it is currently a recommended drug for metastatic ER^+^ breast cancer patients. Methotrexate ranked number 25 (from -1 to 1) and is currently one of the cytotoxic agents for adjuvant chemotherapy. Other compounds were potential drugs for ER^+^ TR patients (Figure S6B). The structures of the top six compounds were acquired from PubChem as previously described (Figure S6C). Although these findings require further investigation, these compounds have shown certain potential for TR breast cancer patients.

## Discussion

The hormone receptor-positive subtype comprises the majority of cases of breast cancer, with more than four-fifths of diagnosed patients exhibiting either ER^+^ or PR^+^ statuses or both 55. Being one of the oldest selective estrogen receptor modulators, tamoxifen has been serving as the first line of adjuvant endocrine therapy for primary and metastatic ER^+^ breast cancer patients 56. However, resistance to tamoxifen therapy is possible and other therapeutic agents are required 57.

In our study, expression levels of four down-regulated miRNAs: hsa-miR-1323, hsa-miR-711, hsa-miR-4287, hsa-miR-650, as well as the five miRNAs, including hsa-miR-532-5p, hsa-miR-1180, hsa-miR-152, hsa-miR-578, hsa-miR-128, were upregulated in combined analyses of GSE46823 and GSE83292 datasets. The prediction software of miRNA-targeting genes showed potential genes in interest. In previous literature, hsa-miR-1323 widely regulate cancer progression and radiotherapy effects 58, miR-711 regulates NCI-N87 and SNU-1 cells gastric cancer lines progression by targeting CD44 59, miR-4287 is a critical molecule in osteoarthritis development 60, miR-650 inhibits the progression of glioma by targeting FAM83F 61. The miR-532-5p is reported to promote cancer proliferation and migration by targeting the *RERG* gene (RAS like estrogen-regulated growth inhibitor) and the *EGFR* (epidermal growth factor receptor) gene 62. Similarly, miR-1180 participates in cell proliferation, migration and invasion by targeting the *DGCR5* (DiGeorge syndrome critical region gene 5) gene, along with resistance to apoptosis by activating the nuclear factor-κB (NF-κB) signaling pathway 63, 64. On the other hand, miR-152 is involved in regulating phosphatidylinositol 3-kinase (PI3K)/AKT and extracellular regulated protein kinases (ERK)/NF-κB signaling pathways, while miR-578 is potentially involved in angiogenesis of *BRCA*-related breast cancer 65, 66. The miR-128 serves as an onco-miR to promote cancer progression in general, and is also responsible for cisplatin resistance in gastric cancer patients 67, 68. In addition to previous findings about the significant correlation between expression of specific miRNAs and resposiveness to tamoxifen in hormone-positive breast cancer patients 69-71, our results help to identify miRNAs in interest by more relevant hypotheses.

On the other hand, upregulated mRNAs in TR compared to TS ER^+^ breast cancer patients obtained from the GSE9893 dataset were matched with those from the GSE7378 dataset. We found that multiple oncogenic pathways were linked with tamoxifen resistance in the present study, including WNT, GPCR, RAS, AKT, TGF-β/BMP, Notch, Hedgehog, and death receptor signaling. In particular, mutation of ESR1 gene is a well-known mechanism of tamoxifen resistance and upregulation of WNT signaling is detected in an* in vitro* model of tamoxifen-resistant breast cancer 72, 73.

Activation of RAS/PI3K/AKT signaling blocks tamoxifen-induced anoikis 74. Hypoxia Inducible Factor 1 Subunit Alpha (HIF-1α) or AMP-Activated Protein Kinase (AMPK) pathways induce autophagy and mediates resistance to anoikis 75, 76. Since other pathways may also be involved in tamoxifen resistance, therfore we analyzed the interaction between miRNA-targeting genes and upregulated mRNAs and discovered *GLUL* plays an important role in cancer progression. GLUL protein promotes cell proliferation in breast cancer 77. Suppression of *GLUL* results in drug resistance in cancer cells 78. However, evidence about the roles of *GLUL* in tamoxifen resistance or recurrence of breast cancer is still absent. Meanwhile, through MetaCore and TIMER analysis revealed that “Immune response_IL-4-induced regulators of cell growth, survival, differentiation and metabolism”, and immune-related pathways were correlated with *GLUL* expression in breast cancer patients from TCGA and METABRIC datasets. IL-4 is a pleiotropic cytokine regulating cell differentiation, survival, and proliferation. IL-4 stimulates cell population proliferation through the activation of several Cyclin-dependent kinases (CDKs), which promote the transition of the cell cycle G1/S phase. IL-4 is also important in the regulation of metabolic processes. Specifically, IL-4/JAK/STAT6 signaling enhances the anabolic actions of insulin and is involved in the metabolism process for different types of cells. These shreds of evidence were consistent with our data and *GLUL* may regulate tamoxifen resistance or the recurrence of breast cancer. Meanwhile, in addition to *GLUL*, we also found several potential candidate genes showing high expression in tamoxifen-treated recurrence breast cancer patients, which may serve as ideal targets for the treatment of breast cancer (Figure 6), including ZNF148 79, VPS4A 80, SPN 81, PTPN9 82, APEX 83, RAE1 84, CCT7 85, USP2 86, and RAGA 87.

Through the CMap analysis, we revealed that particular compounds with highly negative correlation is served as potential therapeutic agents for TR breast cancer patients. Fulvestrant and methotrexate are listed as potential drugs for TR patients. Fulvestrant is one of the standard salvage drugs for metastatic ER^+^ breast cancer 88. Methotrexate is a cytotoxic agent and is used in combined regimens for adjuvant chemotherapy. Although new drugs are being developed, methotrexate is still a useful drug for metastatic breast cancer 89. We also detected other compounds. Proton pump inhibitor omeprazole decreased invasion and metastasis of breast cancer cell line. The combination of sulfasalazine and MK-801 also showed antiproliferative properties in EGFR-overexpressing glioma cells 90. MK-801 also has the same effect on the growth of pancreatic tumor xenografts in nude mice 91. Carbachol in combination with low-dose paclitaxel suppresses the proliferation of breast cancer cells *in vitro* 92. The 8-azaguanine is a potential cytotoxic drug with anticancer ability in a prediction model or *in vitro* study 93, 94. Sirolimus (mTOR inhibitor), an allosteric mTORC1 inhibitor, suppresses the growth of breast cancer cells. Sirolimus and everolimus improve the survival of patients with hepatocellular carcinoma 95. Harmine exhibited anticancer properties in breast cancer cell lines 96. Berberine inhibited autophagy by participating in the PTEN/Akt/mTOR pathway by reversing doxorubicin resistance in breast cancer 97. Collectively, our CMap data suggest that these FDA-approved compounds are potential therapeutic drugs for TR breast cancer patients.

## Conclusion

To summarize, through comprehensive bioinformatics analyses of the transcriptome of four datasets of ER^+^ breast cancer patients and further wet-lab validation, our study presented *GLUL* as a remarkable factor that associated with ER^+^ breast cancer. Furthermore, we also identified the immune signaling pathways by the top co-expressed genes with *GLUL* in ER^+^ breast cancer patients developing tamoxifen resistance. Our results provide a better understanding of the molecular mechanisms leading to tamoxifen resistance in patients undergoing treatment. Finally, computational connectivity map-based production of drugs that can serve as alternative treatments for tamoxifen-resistant ER^+^ breast cancer.

## Supplementary Material

Supplementary figures and tables.
