# Supplementary material for: Glutamine synthetase regulates the immune microenvironment and cancer development through the inflammatory pathway
Source: Int J Med Sci. 2023 Jan 1;20(1):35–49. doi: 10.7150/ijms.75625 (PMC9812810; doi:10.7150/ijms.75625)
Supplement: Supplementary file 1 — Supplementary figures and tables. [file ijmsv20p0035s1.pdf]

Supplementary data

Table S1: **Canonical pathways in an Ingenuity Pathway Analysis (IPA). Analysis of predicted genes from common expressed micro (mi)RNAs in tamoxifen-resistant (TR) estrogen receptor-positive (ER^+) breast cancer patients, compared to tamoxifen-sensitive (TS) patients from the intersection of the top 10% expressed miRNAs in the GSE46823 and GSE83292 datasets. The results show potential miRNA-messenger (m)RNA interactions.**

Ingenuity Canonical Pathways	-log(P-value)	Ratio	Molecules
**IL-15 Production**	4.43	0.0783	ERBB3,FER,KDR,NTRK2,NTRK3,PEAK1,PTK2,ROS1,TNK1
**IL-8 Signaling**	4.05	0.0576	BRAF,GNAS,KDR,MAP4K4,MAPK10,MMP2,PIK3CA,PIK3R5,PLD1,PRKD1,PTK2
**Sperm Motility**	3.67	0.0521	ERBB3,FER,GNAS,KDR,NTRK2,NTRK3,PEAK1,PRKD1,PTK2,ROS1,TNK1
**Synaptogenesis Signaling Pathway**	3.44	0.0433	BRAF,CACNB4,CDH18,CLASP2,CTNND1,GRIN2B,NTRK2,PIK3CA,PIK3R5,SNCA,STXBP6,SYT1,SYT14
**Ovarian Cancer Signaling**	3.13	0.0584	BRAF,BRCA1,MMP2,PIK3CA,PIK3R5,PTGS1,SIN3A,TCF4
**Molecular Mechanisms of Cancer**	2.97	0.0368	BMP5,BRAF,BRCA1,CTNND1,GNAS,MAPK10,NAIP,PIK3CA,PIK3R5,PRKD1,PTCH1,PTK2,SIN3A,TCF4
**Endothelin-1 Signaling**	2.95	0.0497	BRAF,ECE1,GNAS,MAPK10,PIK3CA,PIK3R5,PLD1,PRKD1,PTGS1
**Adrenomedullin signaling pathway**	2.76	0.0466	BRAF,GNAS,KCNN3,MAPK10,MMP2,PIK3CA,PIK3R5,PTK2,TTN
**Signaling by Rho Family GTPases**	2.7	0.0424	ARFIP2,CDH18,CIT,DIAPH3,GNAS,MAPK10,PIK3CA,PIK3R5,PLD1,PTK2

**PTEN Signaling**	2.7	0.056	KDR,NTRK2,NTRK3,PIK3CA,PIK3R5,PTK2,SYNJ1
**Human Embryonic Stem Cell Pluripotency**	2.56	0.053	BMP5,GNAS,NTRK2,NTRK3,PIK3CA,PIK3R5,TCF4
**Axonal Guidance Signaling**	2.56	0.0321	ADAMTS12,BMP5,GNAS,MME,MMP2,NTRK2,NTRK3,PIK3CA,PIK3R5,PRKD1,PTCH1,PTK2,ROBO1,SEMA3D,SEMA4F
**Neuropathic Pain Signaling In Dorsal Horn Neurons**	2.55	0.0606	GRIN2B,KCNN3,NTRK2,PIK3CA,PIK3R5,PRKD1
**Glioma Invasiveness Signaling**	2.48	0.0704	MMP2,PIK3CA,PIK3R5,PLG,PTK2
**Paxillin Signaling**	2.42	0.0571	ARFIP2,ITGA7,MAPK10,PIK3CA,PIK3R5,PTK2
**HGF Signaling**	2.36	0.0556	MAP3K2,MAPK10,PIK3CA,PIK3R5,PRKD1,PTK2
**Thyroid Cancer Signaling**	2.3	0.0816	BRAF,NTRK2,NTRK3,TCF4
**Huntington's Disease Signaling**	2.29	0.0396	ARFIP2,GRIN2B,HDAC9,PIK3CA,PIK3R5,PRKD1,REST,SIN3A,SNCA
**Endocannabinoid Developing Neuron Pathway**	2.25	0.0526	BRAF,BRCA1,MAPK10,PAX6,PIK3CA,PIK3R5
**UVB-Induced MAPK Signaling**	2.24	0.0784	MAPK10,PIK3CA,PIK3R5,PRKD1
**Leukocyte Extravasation Signaling**	2.23	0.0419	CTNND1,FER,MAPK10,MMP2,PIK3CA,PIK3R5,PRKD1,PTK2
**HER-2 Signaling in Breast Cancer**	2.21	0.061	ERBB3,MMP2,PIK3CA,PIK3R5,PRKD1
**Germ Cell-Sertoli Cell Junction Signaling**	2.08	0.0432	CTNND1,FER,MAP3K2,MAPK10,PIK3CA,PIK3R5,PTK2

**Reelin Signaling in Neurons**	2.04	0.0476	APBB1,GRIN2B,MAPK10,PDK3,PIK3CA,PIK3R5
**ErbB Signaling**	2.03	0.0549	ERBB3,MAPK10,PIK3CA,PIK3R5,PRKD1
**GNRH Signaling**	2.01	0.0419	CACNB4,GNAS,MAP3K2,MAPK10,MMP2,PRKD1,PTK2
**Role of NFAT in Cardiac Hypertrophy**	2.01	0.0383	CACNB4,GNAS,HDAC9,MAPK10,PIK3CA,PIK3R5,PRKD1,SLC8A1
**NF-κB Signaling**	1.96	0.0409	BRAF,KDR,MAP4K4,NTRK2,NTRK3,PIK3CA,PIK3R5
**Amyotrophic Lateral Sclerosis Signaling**	1.95	0.0526	GLUL,GRIN2B,NAIP,PIK3CA,PIK3R5
**ATM Signaling**	1.95	0.0526	BRCA1,CBX5,MAPK10,TP53BP1,ZEB1
**UVA-Induced MAPK Signaling**	1.91	0.0515	MAPK10,PARP10,PIK3CA,PIK3R5,TIPARP
**Apelin Cardiomyocyte Signaling Pathway**	1.91	0.0515	MAPK10,PIK3CA,PIK3R5,PRKD1,SLC8A1
**Hepatic Fibrosis Signaling Pathway**	1.91	0.0311	ACVR1C,BRAF,KDR,MAPK10,PIK3CA,PIK3R5,PRKD1,PTCH1,PTK2,TCF4,TTN
**CD40 Signaling**	1.9	0.0625	MAPK10,PIK3CA,PIK3R5,PTGS1
**SAPK/JNK Signaling**	1.9	0.051	MAP3K2,MAP4K4,MAPK10,PIK3CA,PIK3R5
**Glutamine Biosynthesis I**	1.85	1	GLUL
**Choline Biosynthesis III**	1.79	0.143	PCYT1B,PLD1
**Neuroinflammation Signaling Pathway**	1.78	0.0327	ACVR1C,GLUL,GRIN2B,MAPK10,NAIP,PIK3CA,PIK3R5,SLC6A1,SNCA

**Pancreatic Adenocarcinoma Signaling**	1.75	0.0467	MAPK10,PIK3CA,PIK3R5,PLD1,SIN3A
**Small Cell Lung Cancer Signaling**	1.74	0.0563	PIK3CA,PIK3R5,PTK2,SIN3A
**Rac Signaling**	1.7	0.0455	ARFIP2,PIK3CA,PIK3R5,PLD1,PTK2
**FcγRIIB Signaling in B Lymphocytes**	1.67	0.0533	CACNB4,MAPK10,PIK3CA,PIK3R5
**Neurotrophin/TRK Signaling**	1.67	0.0533	NTRK2,NTRK3,PIK3CA,PIK3R5
**eNOS Signaling**	1.66	0.0392	GNAS,KDR,NOSTRIN,PIK3CA,PIK3R5,PRKD1
**Fc Epsilon RI Signaling**	1.66	0.0442	MAPK10,PIK3CA,PIK3R5,PRKD1,SYNJ1
**Apelin Endothelial Signaling Pathway**	1.66	0.0442	GNAS,MAPK10,PIK3CA,PIK3R5,PRKD1
**Apelin Pancreas Signaling Pathway**	1.64	0.0698	MAPK10,PIK3CA,PIK3R5
**IL-12 Signaling and Production in Macrophages**	1.64	0.0439	IL23R,MAPK10,PIK3CA,PIK3R5,PRKD1
**Tec Kinase Signaling**	1.63	0.0385	GNAS,MAPK10,PIK3CA,PIK3R5,PRKD1,PTK2
**IL-23 Signaling Pathway**	1.62	0.0682	IL23R,PIK3CA,PIK3R5
**Renin-Angiotensin Signaling**	1.61	0.0431	MAPK10,PIK3CA,PIK3R5,PRKD1,PTK2
**GP6 Signaling Pathway**	1.61	0.0431	COL20A1,PIK3CA,PIK3R5,PRKD1,PTK2
**nNOS Signaling in Neurons**	1.59	0.0667	GRIN2B,PFKM,PRKD1
**Integrin Signaling**	1.58	0.0343	ASAP1,BRAF,ITGA7,PIK3CA,PIK3R5,PTK2,TTN

**LPS-stimulated MAPK Signaling**	1.57	0.05	MAPK10,PIK3CA,PIK3R5,PRKD1
**Prolactin Signaling**	1.57	0.05	NR3C1,PIK3CA,PIK3R5,PRKD1
**IL-4 Signaling**	1.57	0.05	NR3C1,PIK3CA,PIK3R5,SYNJ1
**Ephrin A Signaling**	1.57	0.0652	PIK3CA,PIK3R5,PTK2
**CXCR4 Signaling**	1.56	0.037	GNAS,MAPK10,PIK3CA,PIK3R5,PRKD1,PTK2
**PEDF Signaling**	1.56	0.0494	PIK3CA,PIK3R5,TCF4,ZEB1
**VEGF Family Ligand-Receptor Interactions**	1.54	0.0488	KDR,PIK3CA,PIK3R5,PRKD1

Table S2: Canonical pathways in an Ingenuity Pathway Analysis (IPA). Analysis of common upregulated genes (top 10%) from tamoxifen-resistant (TR) estrogen receptor-positive (ER^+) breast cancer patients in the GSE9893 and GSE7378 datasets, compared to tamoxifen-sensitive (TS) patients.

Abbreviation: ER, estrogen receptor; IPA, ingenuity pathway analysis; miRNA, micro ribonucleic acid; TR, tamoxifen-resistant; TS, tamoxifen-sensitive

**Ingenuity Canonical Pathways**	**-log(*P* value)**	**Ratio**	**Molecules**
Inhibition of ARE-Mediated mRNA Degradation Pathway	5.56	0.069	CNOT2,CNOT3,CNOT8,PPP2CA,PPP2R1A,SFN,TIA1,ZFP36L1
HOTAIR Regulatory Pathway	4.66	0.0519	CDH1,COL1A1,H3-3A/H3-3B,LEF1,RBBP4,RBBP7,ROCK2,TCF3
Protein Kinase A Signaling	3.89	0.0294	CDKN3,GNB1,GYS1,H3-3A/H3-3B,LEF1,MYL6,PHKG2,PTPRF,ROCK2,SFN,TCF3
Cell Cycle Regulation by BTG Family Proteins	3.87	0.114	CCND1,E2F6,PPP2CA,PPP2R1A

Wnt/β-catenin Signaling	3.54	0.0417	CCND1,CDH1,CSNK1A1,LEF1,PPP2CA,PPP2R1A,TCF3
Cyclins and Cell Cycle Regulation	3.51	0.0641	CCNB2,CCND1,E2F6,PPP2CA,PPP2R1A
HIPPO signaling	3.39	0.0602	DLG1,DLG5,PPP2CA,PPP2R1A,SFN
ILK Signaling	3.36	0.0389	CASP3,CCND1,CDH1,LEF1,MYL6,PPP2CA,PPP2R1A
Adipogenesis pathway	3.34	0.0462	BMP7,CDK5,CTBP2,RBBP4,RBBP7,SREBF1
Thyroid Cancer Signaling	3.3	0.0816	CCND1,CDH1,LEF1,TCF3
Molecular Mechanisms of Cancer	3.21	0.0263	BMP7,CASP3,CCND1,CDH1,CDK5,CTNNA1,E2F6,LEF1,RASA1,TCF3
Endocannabinoid Cancer Inhibition Pathway	3.19	0.0432	CASP3,CCND1,CDH1,LEF1,ROCK2,TCF3
Apelin Liver Signaling Pathway	3.02	0.115	COL1A1,COL2A1,COL5A3
Endometrial Cancer Signaling	2.96	0.0667	CCND1,CDH1,CTNNA1,LEF1
Mitotic Roles of Polo-Like Kinase	2.86	0.0625	CCNB2,HSP90AA1,PPP2CA,PPP2R1A
Tight Junction Signaling	2.84	0.037	CTNNA1,MYL6,PPP2CA,PPP2R1A,STX16,VAMP3
Hepatic Fibrosis Signaling Pathway	2.83	0.0254	CASP3,CCND1,COL1A1,COL2A1,COL5A3,LEF1,MYL6,ROCK2,TCF3
PI3K/AKT Signaling	2.71	0.0349	CCND1,GYS1,HSP90AA1,PPP2CA,PPP2R1A,SFN
DNA Methylation and Transcriptional Repression Signaling	2.67	0.0882	H3-3A/H3-3B,RBBP4,RBBP7
Colorectal Cancer Metastasis Signaling	2.58	0.0285	CASP3,CCND1,CDH1,GNB1,LEF1,TCF3,TLR3

Oleate Biosynthesis II (Animals)	2.53	0.182	FADS2,SCD
Intrinsic Prothrombin Activation Pathway	2.5	0.0769	COL1A1,COL2A1,COL5A3
Estrogen Receptor Signaling	2.46	0.0373	CTBP2,H3-3A/H3-3B,HNRNPD,MED23,MED6
Epithelial Adherens Junction Signaling	2.31	0.0345	CDH1,CTNNA1,LEF1,MYL6,TCF3
Neuregulin Signaling	2.24	0.0421	AREG,CDK5,EREG,HSP90AA1
Cell Cycle: G2/M DNA Damage Checkpoint Regulation	2.22	0.0612	CCNB2,CKS2,SFN
Sumoylation Pathway	2.2	0.0412	CDH1,CTBP2,RAN,UBA2
Cell Cycle Control of Chromosomal Replication	2.13	0.0566	CDK5,MCM2,MCM4
Glutamine Biosynthesis I	2.12	1	GLUL
Huntington's Disease Signaling	2.12	0.0264	CASP3,CDK5,GNB1,RASA1,STX16,VAMP3
Telomerase Signaling	2.11	0.0388	DKC1,HSP90AA1,PPP2CA,PPP2R1A
Role of CHK Proteins in Cell Cycle Checkpoint Control	2.08	0.0545	E2F6,PPP2CA,PPP2R1A
Unfolded protein response	2.08	0.0545	AMFR,INSIG1,SREBF1
Semaphorin Signaling in Neurons	2.06	0.0536	CDK5,PLXNB1,ROCK2
DNA damage-induced 14-3-3σ Signaling	2.05	0.105	CCNB2,SFN

Axonal Guidance Signaling	2.05	0.0193	BMP7,CDK5,GNB1,KLC1,MYL6,PLXNB1,RASA1,ROCK2,SRGAP2
LXR/RXR Activation	2.02	0.0364	APOE,SCD,SREBF1,TLR3
Atherosclerosis Signaling	1.99	0.0357	APOE,COL1A1,COL2A1,COL5A3
Hepatic Fibrosis / Hepatic Stellate Cell Activation	1.94	0.0279	COL1A1,COL2A1,COL5A2,COL5A3,MYL6
GP6 Signaling Pathway	1.94	0.0345	COL1A1,COL2A1,COL5A2,COL5A3
Role of Wnt/GSK-3β Signaling in the Pathogenesis of Influenza	1.89	0.0462	CSNK1A1,LEF1,TCF3
Senescence Pathway	1.85	0.0231	CCNB2,CCND1,E2F6,PPP2CA,PPP2R1A,ZFP36L1
Protein Ubiquitination Pathway	1.84	0.0229	AMFR,HSP90AA1,PSMB2,TRAP1,UBE2C,UBE2G1
Epoxysqualene Biosynthesis	1.82	0.5	SQLE
Spermine Biosynthesis	1.82	0.5	AMD1
Spermidine Biosynthesis I	1.82	0.5	AMD1
Adenine and Adenosine Salvage I	1.82	0.5	APRT
Basal Cell Carcinoma Signaling	1.82	0.0435	BMP7,LEF1,TCF3
Breast Cancer Regulation by Stathmin1	1.81	0.0259	E2F6,GNB1,PPP2CA,PPP2R1A,ROCK2
Gα12/13 Signaling	1.8	0.0315	CDH1,MYL6,RASA1,ROCK2
Estrogen-mediated S-phase Entry	1.79	0.0769	CCND1,E2F6

Hypoxia Signaling in the Cardiovascular System	1.74	0.0405	HSP90AA1,UBE2C,UBE2G1
Osteoarthritis Pathway	1.73	0.0248	CASP3,COL2A1,CTNNA1,LEF1,TCF3
Role of Osteoblasts, Osteoclasts and Chondrocytes in Rheumatoid Arthritis	1.64	0.0235	BMP7,COL1A1,CSNK1A1,LEF1,TCF3
CTLA4 Signaling in Cytotoxic T Lymphocytes	1.63	0.037	AP2M1,PPP2CA,PPP2R1A
Regulation of eIF4 and p70S6K Signaling	1.59	0.027	EIF4G2,PAIP1,PPP2CA,PPP2R1A
Synaptogenesis Signaling Pathway	1.58	0.02	AP2M1,APOE,CDH1,CDK5,STX16,VAMP3
Role of Macrophages, Fibroblasts and Endothelial Cells in Rheumatoid Arthritis	1.57	0.0199	CCND1,CSNK1A1,LEF1,ROCK2,TCF3,TLR3
Factors Promoting Cardiogenesis in Vertebrates	1.54	0.0341	BMP7,LEF1,TCF3
Acute Myeloid Leukemia Signaling	1.54	0.0341	CCND1,LEF1,TCF3
Prostate Cancer Signaling	1.5	0.033	CCND1,HSP90AA1,LEF1

Table S3: Pathway analysis of genes co-expressed with *GLUL* (glutamate-ammonia ligase) from public breast cancer databases using the MetaCore database (with $p<0.01$ set as the cutoff value)

#	Maps	*P* value	Network Objects from Active Data

**1**	Cell cycle_Role of APC in cell cycle regulation	3.66E-10	MAD2b, Tome-1, Cyclin A, Aurora-B, CDC25A, Cyclin B, BUB1, Emi1, Aurora-A, PLK1, CDC20, Securin
**2**	Cell cycle_Cell cycle (generic schema)	1.53E-08	Cyclin A, CDC25A, Cyclin B, E2F2, E2F4, CDK4, Cyclin E, E2F3, CDC25B
**3**	Immune response_IL-4-induced regulators of cell growth, survival, differentiation and metabolism	1.81E-07	STAT3, MCM6, GATA-3, Cyclin A, CISH, CDC25A, MCM5, CDK4, PLEKHF1, SK4/IK1, Cyclin E, Cathepsin V, Bcl-2
**4**	Cell cycle_The metaphase checkpoint	2.91E-07	MAD2b, Aurora-B, HEC, Survivin, CENP-E, BUB1, CENP-A, Aurora-A, PLK1, CDC20
**5**	Cell cycle_Role of SCF complex in cell cycle regulation	4.91E-06	Skp2/TrCP/FBXW, CDC25A, CDK4, SKP1, Emi1, Cyclin E, PLK1, beta-TrCP
**6**	Main genetic and epigenetic alterations in lung cancer	5.14E-06	DNMT3B, GSTP1, IGF-1 receptor, MSP, TGF-alpha, IBP4, CDK4, IBP, Semaphorin 3B, Bcl-2
**7**	Ligand-independent activation of Androgen receptor in Prostate Cancer	1.95E-05	STAT3, Androgen receptor, IGF-1 receptor, PP2A regulatory, PP2A catalytic, FRS2, SRD5A1, IRS-1, Frizzled, Prolactin receptor, ErbB3
**8**	Cell cycle_ESR1 regulation of G1/S transition	2.23E-05	Cyclin A, Skp2/TrCP/FBXW, CDC25A, E2F4, ESR1 (nuclear), CDK4, Cyclin A2, Cyclin E
**9**	Development_Positive regulation of WNT/Beta-catenin signaling in the nucleus	2.59E-05	Casein kinase II, alpha chains, SOX11, BCL9/B9L, APPL, TLE, HDAC2, RUNX, Casein kinase I alpha, beta-TrCP, Frizzled, FOXM1
**10**	Development_Negative regulation of WNT/Beta-catenin signaling in the cytoplasm	2.73E-05	KLHL12, WWP1, Skp2/TrCP/FBXW, PP2A catalytic, CXXC5, Nucleoredoxin, RIPK4, Casein kinase I alpha, Amer1, beta-TrCP, Frizzled, Beclin 1
**11**	PR action in breast cancer: stimulation of cell growth and proliferation	2.79E-05	STAT3, ESR1 (membrane), Cyclin A, ESR1 (nuclear), Cyclin E, PR (membrane), PR (nuclear), ErbB3
**12**	Cell cycle_Regulation of G1/S transition (part 1)	4.23E-05	PP2A regulatory, Cyclin A, Skp2/TrCP/FBXW, CDC25A, PP2A catalytic, CDK4, Cyclin E, beta-TrCP
**13**	Abnormalities in cell cycle in SCLC	5.05E-05	Cyclin A, Aurora-B, E2F2, CDK4, Cyclin E, E2F3, BMI-1

**14**	Cell cycle progression in Prostate Cancer	5.16E-05	STAT3, Androgen receptor, CDC25A, Cyclin B, CDK4, Frizzled, CDC25B, Prolactin receptor
**15**	Reproduction_Progesterone-mediated oocyte maturation	6.26E-05	ESR1 (membrane), BUB1, Aurora-A, PLK1, Adenylate cyclase, CDC20, CDC25B, PR (membrane)
**16**	Cell cycle_Chromosome condensation in prometaphase	6.32E-05	Cyclin A, Aurora-B, Cyclin B, CAP-G, CAP-G/G2, Aurora-A
**17**	Apoptosis and survival_BAD phosphorylation	9.03E-05	IGF-1 receptor, Adenylate cyclase type I, PP2C, PP2A catalytic, IRS-1, Bcl-2, Beclin 1, 14-3-3
**18**	Cell cycle_Spindle assembly and chromosome separation	1.22E-04	Aurora-B, HEC, Tubulin alpha, Cyclin B, Aurora-A, CDC20, Securin
**19**	Immune response_IL-11 signaling via JAK/STAT	1.49E-04	STAT3, gp130, Survivin, Pim-1, Bcl-2, RARalpha, CYP19
**20**	Higher ESR1 / ESR2 ratio in breast cancer	2.18E-04	CDC25A, BTG2, ESR1 (nuclear), Cyclin A2, Cyclin E, FOXM1, ErbB3
**21**	Pro-oncogenic action of Estradiol/ Estrogen receptors in ovarian cancer	2.27E-04	STAT3, ESR1 (membrane), Survivin, ESR1 (nuclear), TFF1, Cyclin A2, 14-3-3 theta, Cyclin E, MEK4(MAP2K4)
**22**	DNA damage_ATM / ATR regulation of G2 / M checkpoint	3.56E-04	Cyclin A, Cyclin B, Cyclin B2, BLM, ATRIP, CDC25B
**23**	IL-6 signaling in breast cancer cells	4.84E-04	STAT3, gp130, MUC1, Survivin, ESR1 (nuclear), Bcl-2, Fascin, CYP19
**24**	Breast cancer (general schema)	5.05E-04	Androgen receptor, ESR1 (membrane), ESR1 (nuclear), PR (membrane), Prolactin receptor, PR (nuclear), ErbB3
**25**	Role of ER stress in obesity and type 2 diabetes	5.51E-04	SREBP1 precursor, ATF-6 alpha (90kDa), SREBP1 (nuclear), BI-1, SREBP1 (Golgi membrane), IRS-1, XBP1, ATF-6 alpha (50kDa)
**26**	Epigenetic alterations in ovarian cancer	5.79E-04	DNMT3B, GSTP1, HRK, Aurora-B, BLU, HDAC2, ESR1 (nuclear), CDK4, Aurora-A, CDC20

**27**	DNA damage_ATM/ATR regulation of G1/S checkpoint	7.60E-04	Cyclin A, CDC25A, CDK4, BLM, Cyclin E, ATRIP
**28**	Cell cycle_Start of DNA replication in early S phase	7.60E-04	ORC6L, PP2A catalytic, MCM5, Cyclin E, MCM10, CDC45L
**29**	Prolactin signaling in Prostate Cancer	9.02E-04	Androgen receptor, PIP, PDH, PDH alpha, PDHA (somatic), Prolactin receptor
**30**	Pro-oncogenic action of Androgen receptor in breast cancer	1.25E-03	Androgen receptor, PIP, MUC1, SRD5A1, HNF3-alpha, ErbB3
**31**	Prolactin/ JAK2 signaling in breast cancer	1.28E-03	STAT3, CISH, SK4/IK1, c-Myb, Prolactin receptor
**32**	Development_Positive regulation of WNT/Beta-catenin signaling in the cytoplasm	1.34E-03	Casein kinase II, alpha chains, IGF-1 receptor, Bcl-9, PP2A catalytic, SIAH2, IRS-1, RIPK4, Frizzled, 14-3-3
**33**	Regulation of CFTR activity (normal and CF)	1.40E-03	Casein kinase II, alpha chains, PDZK1, PP2A regulatory, PP2C, PP2A catalytic, Filamin B (TABP), AMPK beta subunit, Adenylate cyclase
**34**	Immune response_IL-3 signaling via JAK/STAT, p38, JNK and NF-kB	1.55E-03	STAT3, CISH, Survivin, Cyclin A2, XBP1, Pim-1, IKK-beta, Bcl-2, MEK4(MAP2K4), RARalpha
**35**	IGF-1 receptor/EGFR cooperation in lung cancer	1.56E-03	IGF-1 receptor, Amphiregulin, Survivin, IRS-1, 14-3-3 theta
**36**	IL-6 signaling in colorectal cancer	1.68E-03	STAT3, gp130, Cyclin B, Survivin, TFF3, Cyclin E
**37**	Cell cycle_Initiation of mitosis	1.87E-03	Lamin B, Cyclin B2, PLK1, CDC25B, FOXM1
**38**	IL-6 signaling in Prostate Cancer	2.54E-03	STAT3, Androgen receptor, gp130, MCP, IRS-1, Pim-1
**39**	Cell cycle_Regulation of G1/S transition (part 2)	2.64E-03	Cyclin A, E2F4, CDK4, Cyclin A2, Cyclin E
**40**	Stem cells_Inhibition of Hedgehog signaling in medulloblastoma stem cells	2.89E-03	Adenylate cyclase type I, FRS2, FGFR4, BMI-1, DOCK1, MEK4(MAP2K4)

**41**	Immune response_IL-5 signaling via JAK/STAT	3.32E-03	STAT3, Amphiregulin, CISH, Survivin, XBP1, Pim-1, Bcl-2
**42**	Transcription_Ligand-dependent activation of the ESR1/SP pathway	3.62E-03	CDC25A, ESR1 (nuclear), Cyclin E, RARalpha, Prolactin receptor
**43**	Development_WNT/Beta-catenin signaling pathway. Signalosome	3.69E-03	Casein kinase I gamma 1, PP2A catalytic, GRK6, Casein kinase I alpha, Amer1, Frizzled
**44**	Anti-apoptotic pathways in endoplasmic reticulum stress response in multiple myeloma	3.84E-03	ATF-6 alpha (90kDa), XBP1, ATF-6 alpha (50kDa), Bcl-2
**45**	Development_Negative regulation of WNT/Beta-catenin signaling in the nucleus	3.99E-03	ZNF703, KDM2, BCL9/B9L, TLE, HDAC2, NARF, RUNX3, Frizzled, 14-3-3
**46**	Putative role of Estrogen receptor and Androgen receptor signaling in progression of lung cancer	4.05E-03	Androgen receptor, ESR1 (membrane), SRD5A1, ESR1 (nuclear), Bcl-2, CYP19, 14-3-3
**47**	Signal transduction_Leptin signaling in non-neuronal cells	4.05E-03	Casein kinase II, alpha chains, STAT3, PPAR-beta(delta), SUR1, Kir6.2, IRS-1, Bcl-2
**48**	EGFR family signaling in pancreatic cancer	4.71E-03	STAT3, TGF-alpha, Survivin, CDK4, Cyclin E, Bcl-2, MEK4(MAP2K4), ErbB3
**49**	Cell cycle_Role of Nek in cell cycle regulation	4.83E-03	HEC, Tubulin alpha, IRS-1, Nek11, Aurora-A
**50**	Signal transduction_mTORC1 downstream signaling	4.90E-03	STAT3, HMGCS2, SREBP1 (nuclear), LIPIN1, IRS-1, PDCD4, PFKP

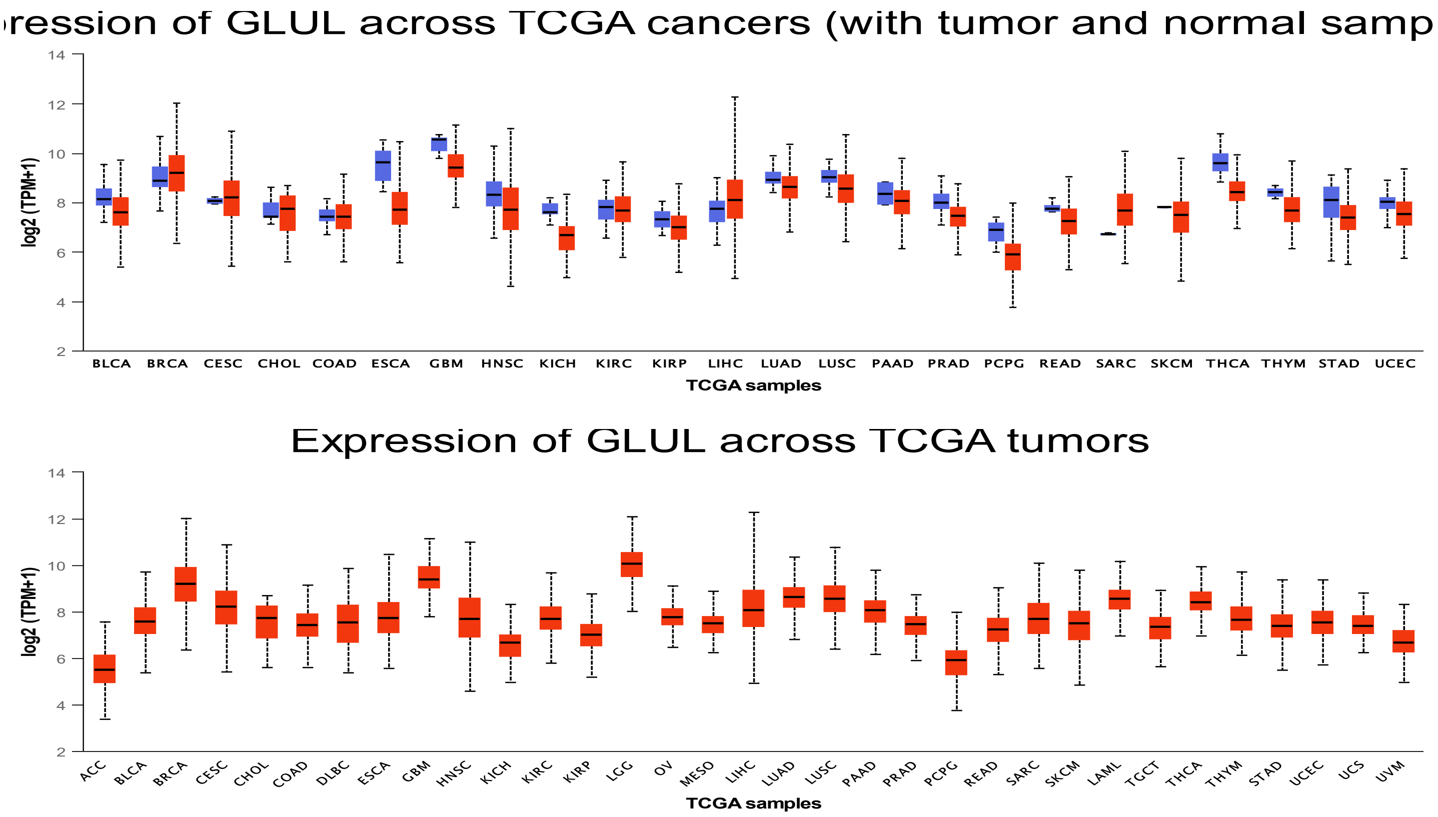

Introduction to Cancer terms

Cancer	TCGA code
Adrenocortical carcinoma	ACC
Bladder urothelial carcinoma	BLCA
Brain lower grade glioma	LGG
Breast invasive carcinoma	BRCA
Cervical squamous cell carcinoma	CESC
Cholangiocarcinoma	CHOL
Colon adenocarcinoma	COAD
Esophageal carcinoma	ESCA
Glioblastoma multiforme	GBM
Head and Neck squamous cell carcinoma	HNSC
Kidney Choromophobe	KICH
Kidney renal clear cell carcinoma	KIRC
Kidney renal papillary cell carcinoma	KIRP
Liver hepatocellular carcinoma	LIHC
Lung adenocarcinoma	LUAD
Lung squamous cell carcinoma	LUSC
Lymphoid Neoplasm Diffuse Large B-cell Lymphoma	DBLC
Mesothelioma	MESO
Ovarian serous cystadenocarcinoma	OV
Pancreatic adenocarcinoma	PAAD
Pheochromocytoma and Paraganglioma	PCPG
Prostate adenocarcinoma	PRAD
Rectum adenocarcinoma	READ
Sarcoma	SARC
Skin Cutaneous Melanoma	SKCM
Testicular Germ Cell Tumors	TGCT
Thymoma	THYM
Thyroid carcinoma	THCA
Uterine Carcinosarcoma	UCS
Uterine Corpus Endometrial Carcinoma	UCEC
Uveal Melanoma	UVM

Figure S1: Differential expressions of glutamate-ammonia ligase (GLUL) between various normal and tumor cells in TCGA database.

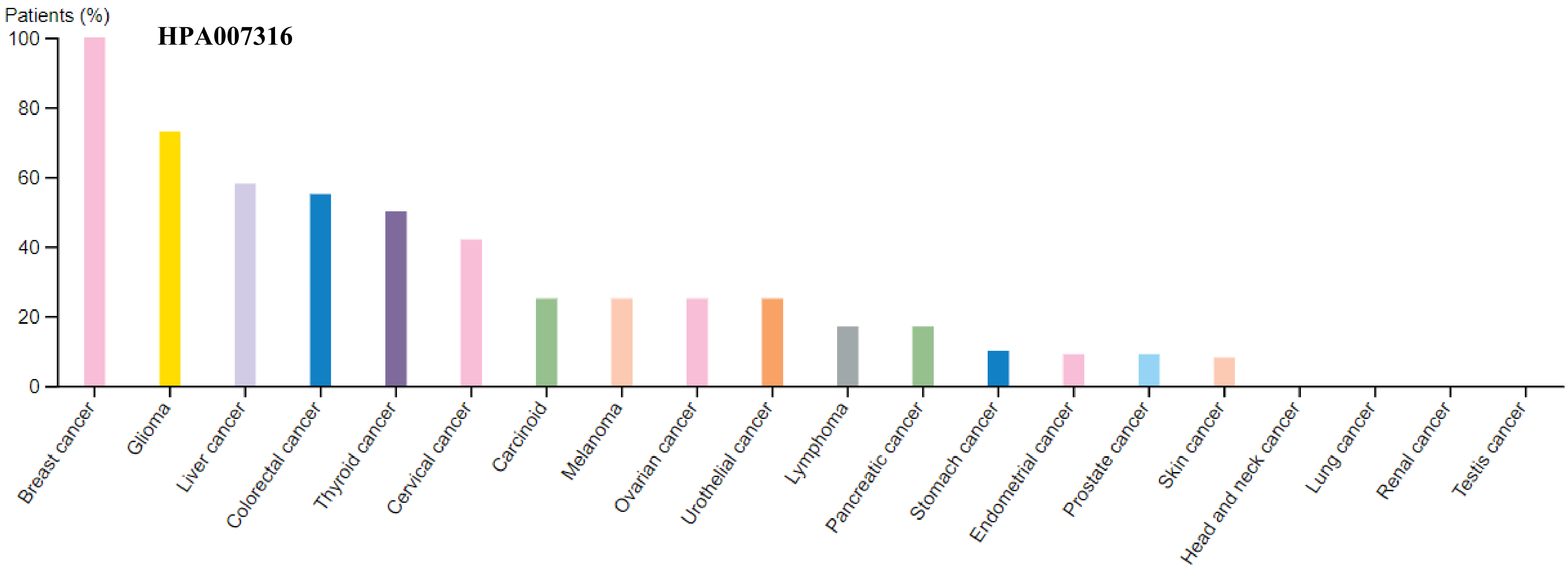

Figure S2: Differential expressions of glutamate-ammonia ligase (GLUL) between various normal and tumor cells in the Proteinatlas database.

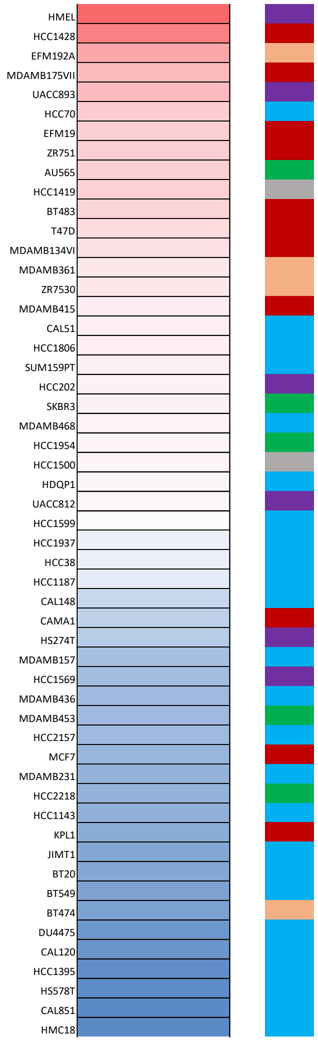

Classification		Immunoprofile		
		ER	PR	HER2
	Luminal A	+	+/−	−
	Luminal B	+	+/−	+
	HER2	−	−	+
	TNBC	−	−	−
	Inconsistent			
	Others			

Figure S3: Heatmap plots showing the glutamate-ammonia ligase (GLUL) expression status in a breast cancer cell line (CCLE), with colored columns on the right side displaying the molecular subtype of each cell line.

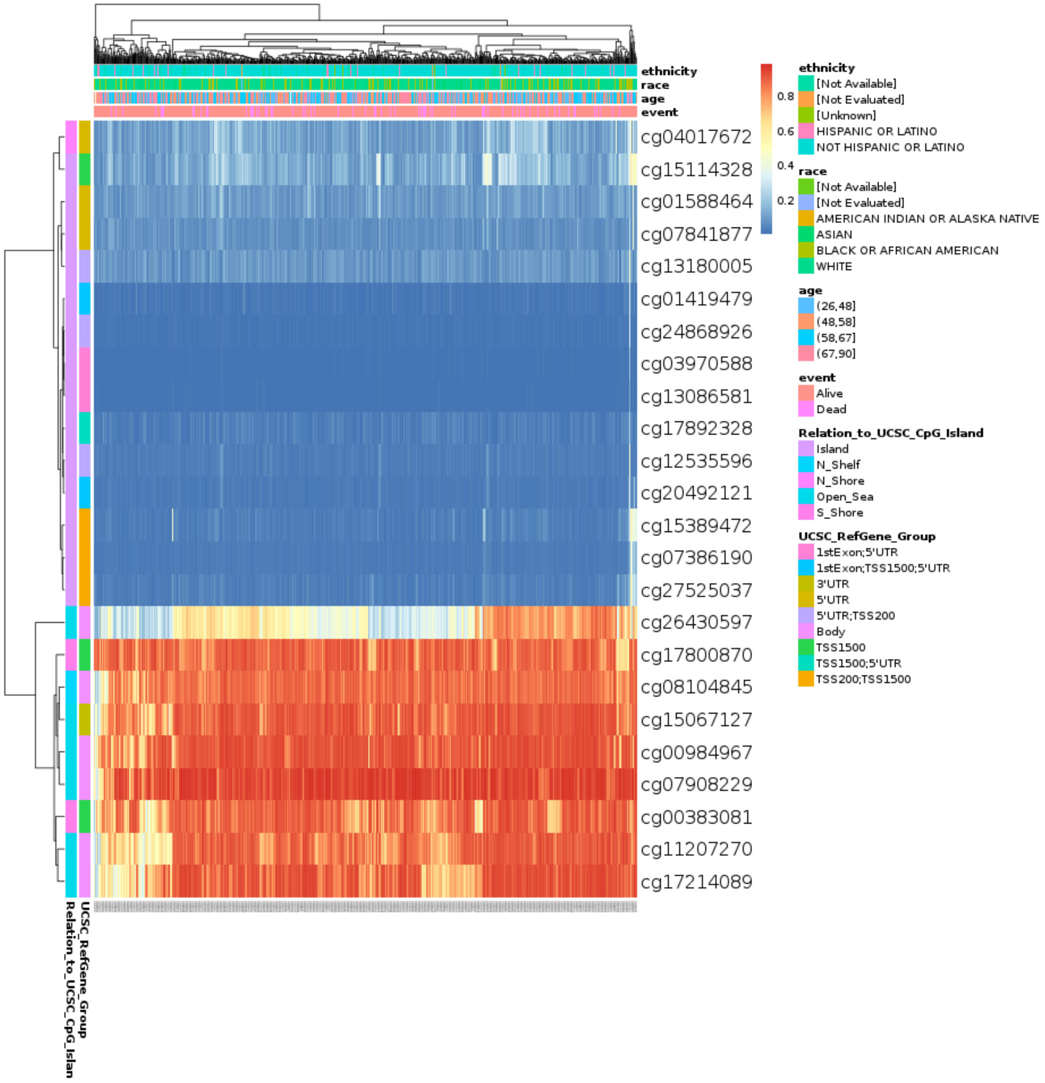

Figure S4: Heatmap of DNA methylation expression levels of glutamate-ammonia ligase (GLUL) in breast cancer by the MethSurv platform.

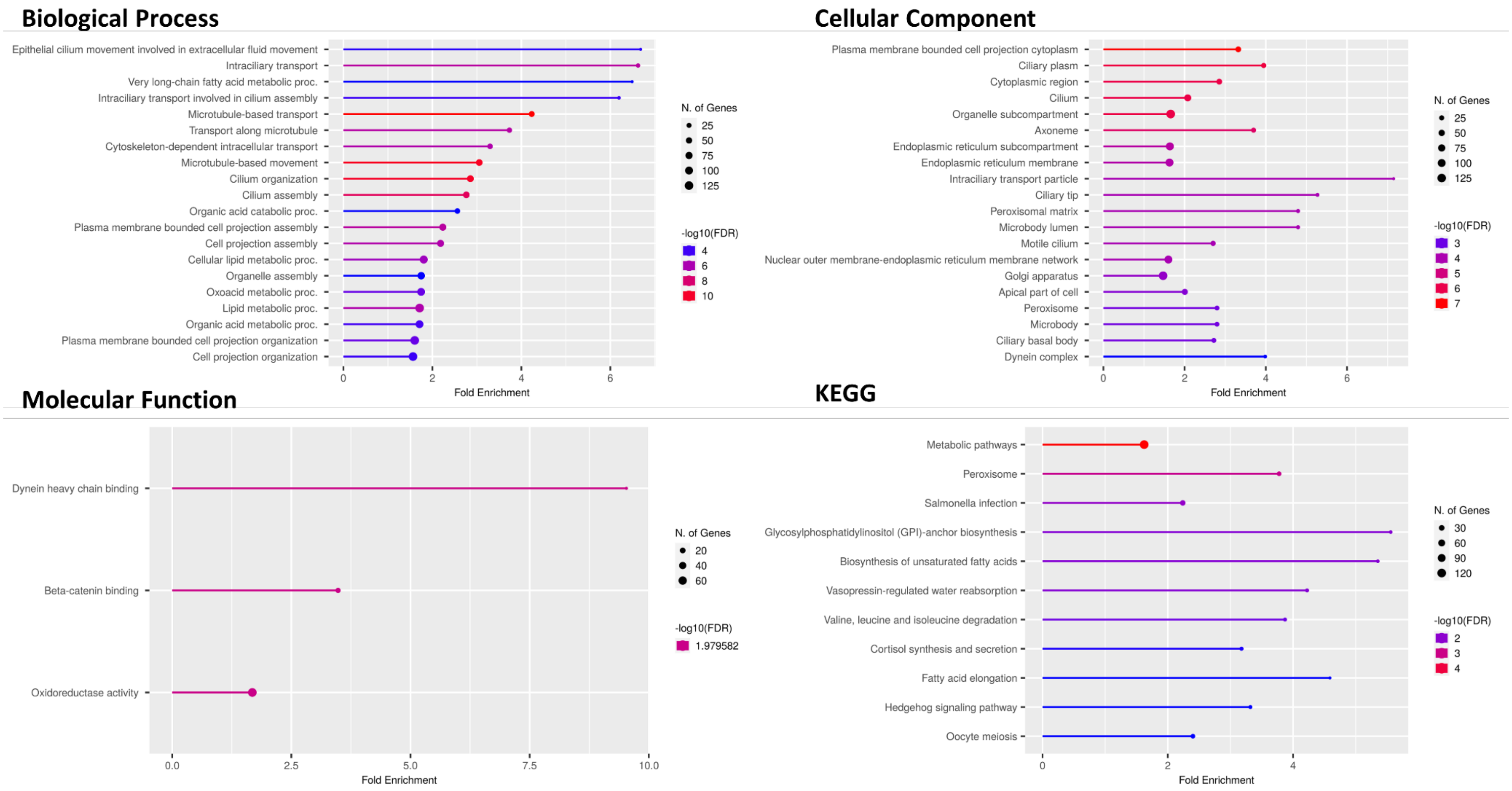

Figure S5: Gene ontology (GO) and Kyoto Encyclopedia of Genes and Genomes (KEGG) analysis based on genes co-expressed with *GLUL* (glutamate-ammonia ligase) from associated TCGA datasets.

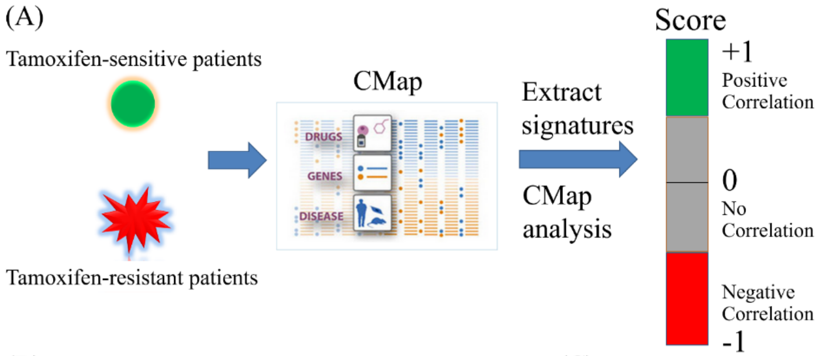

(B)

Rank (+1 → -1)	Rank (-1 → +1)	CMap name	Dose	Cell	Score
6100	1	omeprazole	12 µM	MCF7	-1
6099	2	methacholine chloride	20 µM	MCF7	-0.982
6098	3	ioversol	5 µM	MCF7	-0.977
6097	4	fulvestrant	1 µM	MCF7	-0.974
6096	5	difenidol	12 µM	MCF7	-0.972
6095	6	cycloserine	39 µM	MCF7	-0.965
6094	7	(-)-MK-801	12 µM	MCF7	-0.957
6093	8	carbachol	22 µM	MCF7	-0.955
6092	9	8-azaguanine	26 µM	MCF7	-0.948
6091	10	hydroxyachillin	14 µM	MCF7	-0.941
6089	11	sirolimus	100 nM	MCF7	-0.935
6090	12	mifepristone	9 µM	MCF7	-0.935
6088	13	trazodone	10 µM	MCF7	-0.934
6087	14	desoxycortone	12 µM	MCF7	-0.933
6086	15	clorgiline	13 µM	MCF7	-0.932
6085	16	scopolamine	12 µM	MCF7	-0.928
6084	17	berberine	11 µM	MCF7	-0.922
6081	18	solanine	5 µM	MCF7	-0.921
6082	19	harmine	16 µM	MCF7	-0.921
6083	20	talampicillin	8 µM	MCF7	-0.921
6080	21	protoveratrine A	5 µM	MCF7	-0.917
6076	22	trichostatin A	100 nM	MCF7	-0.914
6077	23	tretinoin	1 µM	MCF7	-0.914
6078	24	midecamycin	5 µM	MCF7	-0.914
6079	25	methotrexate	9 µM	MCF7	-0.914

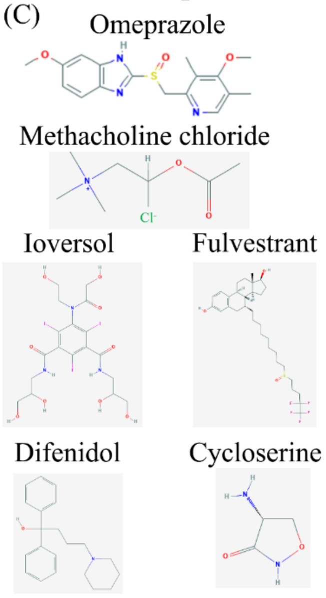

Figure S6: Potential compounds for estrogen receptor-positive (ER^+) tamoxifen-resistant (TR) breast cancer patient treatment, predicted by Connectivity Map (CMap)and ranked in increasing order of negative scores.

(A) Flowchart of the CMap analyses. (B) List of the top 25 compounds with most negative correlations to ER^+ TR breast cancer, listed in increasing order of scores. (C) Chemical structures of six drugs with the most potential to cope with the TR status in hormone-positive breast cancer, as predicted by CMap.
